# Emerging roles of protein modifications in sexual reproduction and pathogenesis of filamentous fungi

**DOI:** 10.3389/fmicb.2026.1744933

**Published:** 2026-01-26

**Authors:** Xiaoxing Li, Xin Zhou, Yuchen Luo, Luman Xue, Xinxin Tong, Jinlin Guo

**Affiliations:** The Ministry of Education Key Laboratory of Standardization of Chinese Medicine, The Lab for Innovation & Effective Uses of Chinese Drug Germplasm Resources, School of Pharmacy, Chengdu University of Traditional Chinese Medicine, Chengdu, Sichuan, China

**Keywords:** filamentous fungi, fungal pathogenicity, post-translational modification, PTMs crosstalk, sexual development

## Abstract

Posttranslational modifications play pivotal roles in the regulation of protein function, enabling precise and dynamic control of diverse cellular processes in fungi. Classical and emerging PTMs, such as phosphorylation, ubiquitination, acetylation, lysine succinylation, and SUMOylation, glycosylation, lipidation modifications, S-acylation, or S-palmitoylation, critically modulate the activity and behavior of proteins. In recent years, research efforts have increasingly focused on global PTMs profiling and functional characterization across fungal species. PTMs function in multiple cellular processes, such as meiosis, cell wall integrity, autophagy, reactive oxygen species metabolism, RNA editing, finely regulating the fungal sexual development and virulence. More recently, the biomolecular condensates dynamics resembled by PTMs modulate host–pathogen interactions. Furthermore, the crosstalk between different PTMs on a single protein and interacting proteins allows for sophisticated regulatory control over fungal development, adaptation, and pathogenicity. However, the full scope of PTMs in the fungal sexual development and pathogenesis in plant remains to be fully elucidated. This review offers a comprehensive analysis of the roles of PTMs in sexual development of the model and plant pathogenic filamentous fungi. It offers mechanistic insights into how the PTMs regulate biological processes, cellular functions and integrate environmental cues, ultimately modulating sexual progression and virulence. A deeper understanding of the roles and regulatory mechanisms of PTMs will facilitate the development of effective strategies for industrially valuable fungi breeding and plant diseases control.

## 1 Introduction

Protein post-translational modifications (PTMs) are covalent processes that proteins undergo during or after translation. They involve the addition of a modifying group to one or several amino acid residues or the removal of a group from the protein by hydrolysis (Suskiewicz, 2024). Common PTMs such as phosphorylation (PM), ubiquitination (UM), acetylation (AM), and SUMOylation can alter the physicochemical properties of proteins, thereby regulating their solubility, interaction, subcellular localization, and activity. By acting a coordinate manner, different PTMs integrate to regulate biological processes (Retanal et al., 2021). The PTMs of the fungal proteins have shown to be extensively involved in multiple physiological and biochemical processes, such as hyphal growth and sexual development, environmental cues adaption and fungal pathogenesis.

Sexual development is a critical phase in the fungal life cycle, including primordium initiation, fruiting body morphogenesis, ascoma differentiation, ascus development, and ascospore maturation (Pöggeler et al., 2018; Debuchy et al., 2010). Fungi rely on mating types of genes that are determined by allelic differences at a specialized chromosomal region called the mating-type locus (*MAT*). Yeast mating and invasion pathways converge on the conserved transcription factor (TF) Ste12, which recruits distinct cofactors to activate mating- or invasion-specific genes. Orthologous pathways are widespread among filamentous fungi. In *Aspergillus nidulans*, Ste12 phosphorylation by the Fusion 3 (Fus3) MAP kinase regulates MAT gene expression (Bayram et al., 2012). Homologs of the yeast Fus3/Kss1 mitogen-activated protein kinase (MAPK) pathway and its target Ste12-like protein are essential for host cuticle penetration and pathogenicity in numerous ascomycete pathogens (Hoang et al., 2024).

Fungal pathogens such as *Sclerotinia sclerotiorum* and *Fusarium graminearum* generates ascospores that serve as the primary inoculum for crop infection (Niu et al., 2024; Shang et al., 2024). Sexual reproduction is also crucial for the infection of *F. graminearum* and is indispensable for the emergence and dissemination of wheat *Fusarium* head blight (FHB) (Niu et al., 2024). To successfully invade and colonize their hosts, these fungi have evolved sophisticated, multi-step infection strategies (Fukada, 2024). This process is mediated by conserved signaling pathways. For instance, MAPK pathways, including fusion 3/kinase suppressor of sst21 (Fus3/Kss1), suppressor of lytic defect 2 (Slt2), and high osmolarity glycerol response protein kinase 1 (Hog1), are critical for virulence in numerous fungal pathogens (Lengeler et al., 2000). These signaling pathways are precisely regulated by higher-order complexes (Lengeler et al., 2000). A key example is the striatin-interacting phosphatase and kinase (STRIPAK) complex, an evolutionarily conserved supramolecular assembly with a critical role in protein (de) phosphorylation (Reschka et al., 2018). In *F. graminearum*, the STRIPAK complex interacts with Mgv1, a key component of the cell wall integrity (CWI) pathway, to regulate fungal development and virulence, illustrating that multi-tiered regulatory networks ultimately fine-tune the pathogenic process (Hou et al., 2002). During infection process, fungal pathogens secrete effector proteins to suppress host immunity and promote colonization. For example, the chitin-binding effector Slp1 of *Magnaporthe oryzae* requires N-glycosylation at three specific sites to fully inhibit chitin-triggered immunity (Chen X. L. et al., 2014). These effectors play pivotal roles in modulating host defense responses, presenting them as promising targets for novel disease control strategies. Moreover, transcriptional regulation, particularly through epigenetic mechanisms like histone modifications (HM), is well-established in shaping pathogenicity (Kramer et al., 2023). Together, PTMs add a critical layer of regulatory complexity to host–pathogen interactions, enabling pathogens to dynamically adapt and counteract host defenses.

Recently, emerging PTMs have been implicated in fungal sexual development and virulence, including lysine succinylation (Ksucc), SUMOylation, glycosylation, lipidation modifications, S-acylation or S-palmitoylation. For instance, knockout of SUMO-related genes results in severe defects in vegetative growth, asexual reproduction, conidial morphology, and spore germination (Azizullah et al., 2023). Moreover, many proteins are simultaneously regulated by multiple types of PTMs, termed PTM crosstalk. The coordinated action of different PTMs acts on the same or interacting proteins to achieve higher order regulatory complexity (Leutert et al., 2021). Through such crosstalk, PTMs integrate diverse signals to fine-tune protein interactions, stability, and localization, effectively serving as signaling hubs that govern multiple biological processes in fungi. For instance, the interaction between plants and pathogenic fungi may involve complex epidermal modifications that exhibit crosstalk with histone acetylation, which plays a role in regulating plant resistance and modulating the development of pathogenic fungi (Zhang X. et al., 2024).

Collectively, PTMs orchestrates the fungal sexual development, pathogenesis, and stress adaptation. This review emphasizes the signaling pathways and mechanistic insights underlying PTMs-mediated regulation. These advances open new avenues for elucidating the molecular mechanisms governing fungal development, stress response, and phytopathogenicity, thereby offering promising strategies for enhancing industrially valued fungi strains breeding and improving biocontrol approaches against plant diseases.

## 2 Sexual development: importance, key pathways, and related proteins

Sexual reproduction represents a pivotal stage in the life cycle of fungi. This process involves the formation of meiotically derived sexual spores, typically developed within multicellular fruiting bodies, and requires precise coordination between the differentiation of diverse cell types and the progression of karyogamy and meiosis. It encompasses several stages, including primordium initiation, fruiting body morphogenesis, ascoma differentiation, ascus development, and ascospore maturation (Pöggeler et al., 2018; Debuchy et al., 2010).

**Figure 1:**
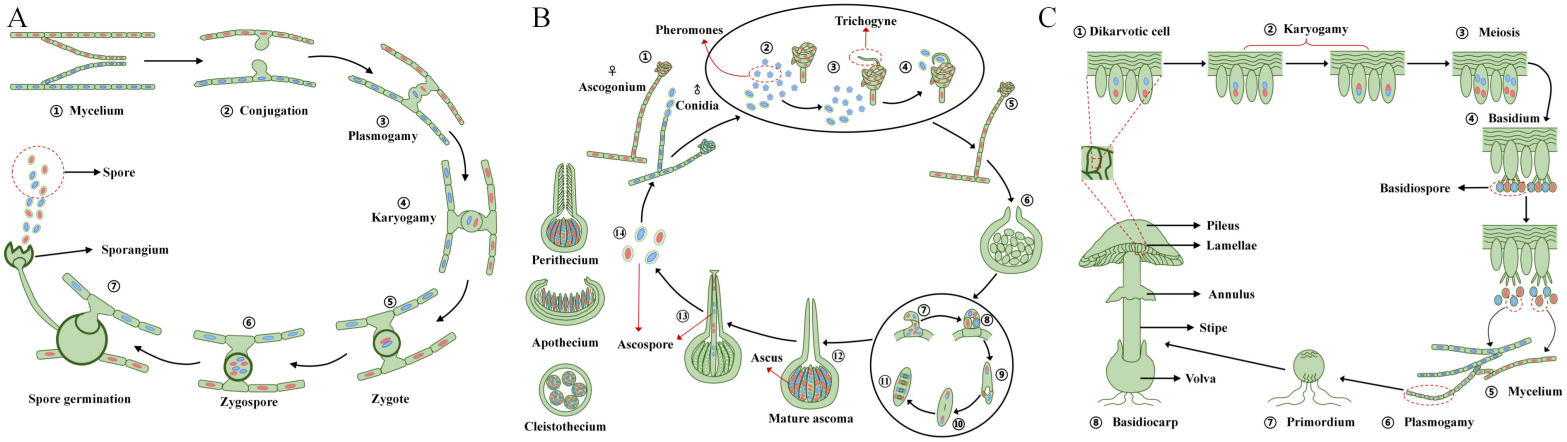
Sexual reproduction in filamentous model fungi of Zygomycota, Ascomycota, and Basidiomycota. **(A)** In Zygomycota, two mating-type hyphae interact, and their tips differentiate into gametangia that fuse through plasmogamy, forming a zygosporangium containing multiple haploid nuclei. The zygosporangium develops a thick wall, followed by karyogamy to produce diploid nuclei. Upon maturation, meiosis generates haploid spores for dispersal. **(B)** In Ascomycota, the male conidium releases pheromones that induce the female ascogonium to produce a receptive trichogyne, which grows toward and fuses with the conidium. After fertilization, the fused cells develop into a protoperithecium, and mature ascospores form within each ascus through meiosis. These mature ascospores are released from fruiting bodies (perithecia, apothecia, or cleistothecia). **(C)** In Basidiomycota, two compatible mycelia fuse to form mononuclear dikaryotic hyphae, four haploid nuclei are produced through meiosis, and these nuclei further develop into basidiospores. The basidiopores germinate into mycelia, the compatible mycelia fuse to form dikaryotic hyphae and further differentiate into primordia that develop into mature basidiocarps.

Reproductive structures exhibit remarkable diversity across taxa, presenting specialized evolutionary adaptations to ecological niches and reproductive strategies (Abrego et al., 2024). In *Mucor* spp., sexual reproduction occurs through gametangia fusion between compatible mating types, forming multinucleate cells that develop into thick-walled zygospores capable of surviving harsh conditions (Figure 1A) (Spatafora et al., 2016). Ascomycota produce complex ascocarps through a regulated developmental process, involving ascogonia-antheridia fusion and crozier formation, with three main morphological types: completely enclosed cleistothecia (e.g., *A. nidulans*) (Sohn and Yoon, 2002), flask-shaped perithecia with apical openings (e.g., *Neurospora crassa*) (Galagan et al., 2003), and cup-shaped apothecia with exposed hymenia (e.g., *Morchella* and *Peziza*) (Figure 1B) (Zhang et al., 2023; Borovička et al., 2024). Basidiomycota develop the most elaborate sexual structures, forming basidiocarps that produce exogenous basidiospores on specialized basidia, with many species maintaining dikaryotic hyphae through clamp connections (e.g., *Lentinula edodes, Tremella fuciformis*, and *Ganoderma lucidum*) (Figure 1C) (Virágh et al., 2022; Raudaskoski, 2015). These diverse sexual structures reflect evolutionary adaptations to ecological niches, reproductive strategies.

Secondary metabolites are linked to sexual development in fungi. For instance, in *Aspergillus flavus*, increased biosynthesis of aflavazole and aflavinine-related compounds promotes the formation of sexual structures. Furthermore, genes in secondary metabolism gene clusters 30 and 44 may be involved in sexual structure formation (Luis et al., 2022). Understanding these genetic and metabolic regulators inform the design of valuable strategies against aflatoxigenic *A. flavus* strains and novel biocontrol agents. Similarly, in *Penicillium chrysogenum*, the MAT1-1-1 TF regulates penicillin biosynthesis, hyphal morphogenesis, and conidiation (Böhm et al., 2013).

To date, over 100 proteins have been identified as critical regulators of sexual development in model fungi, functioning across key processes such as *MAT* loci determination, signal transduction via conserved complexes (MAPK, NADPH Oxidase (NOX), STRIPAK), autophagy, transcriptional/chromatin regulation, and RNA editing (Sun et al., 2023; Varga et al., 2014; Wang et al., 2022). Studies on *S. macrospora* has revealed essential developmental genes (e.g., *acl1, pro1, pro11*), with proline-rich protein 1 (PRO1) and proline-rich protein 11 (PRO11) acting as TFs that coordinate fruiting body development by integrating signaling from the STRIPAK, CWI-MAPK, and NADPH oxidase pathways (Teichert et al., 2020). MAPK cascades, exemplified by the Fus3 module in *Saccharomyces cerevisiae* and its homologs in *A. nidulans*, coordinate sexual development with secondary metabolism (Bayram et al., 2012). NOX complexes (e.g., NADPH Oxidase 1 (NOX1) in perithecia maturation, NOX2 in ascospore germination) fine-tune developmental progression via ROS signaling in *S. macrospora* (Dirschnabel et al., 2014). Hence, the expanding repertoire of sexual developmental proteins with insights into PTM regulatory mechanisms, is enabling an increasingly detailed and systematic understanding of the molecular basis underlying fruiting body development.

## 3 Classical PTMs

### 3.1 Protein phosphorylation modification

PM is the most common PTM type. Protein kinases and phosphatases mediate cellular homeostasis by the continual adjustment of complex signal transduction events in response to various internal and external environmental cues. PM is a reversible modification that is crucial for the regulation of diverse cellular processes, including metabolism, cell cycle, transcription, mating, cell wall synthesis, maintenance of cellular integrity in stress situations (e.g., high-osmolarity and heat stresses), and virulence. In eukaryotic cells, these amino acids are typically Ser (S), Thr (T), and Tyr (Y). Phosphorylation-dephosphorylation cycles serve as “on-off” switches that can trigger conformational changes of target proteins and alter their properties.

#### 3.1.1 MAPK cascades

MAPK cascades are evolutionarily conserved signaling modules that function as central signal transduction systems in eukaryotes, converting extracellular stimuli into coordinated intracellular responses to regulate fundamental processes. In fungi, a sequential phosphorylation relays a MAPK kinase kinase (MAPKKK), a MAPK kinase (MAPKK), and a MAPK, governing key biological functions including mating, osmoadaptation, CWI, hyphal morphogenesis, sporulation, and pathogenicity. Major MAP kinase cascades encompass the pheromone response (PR) pathway, CWI pathway, and the osmosensing pathway. The inhibition of the PR pathway blocks mating and fruiting body formation.

**Figure 2:**
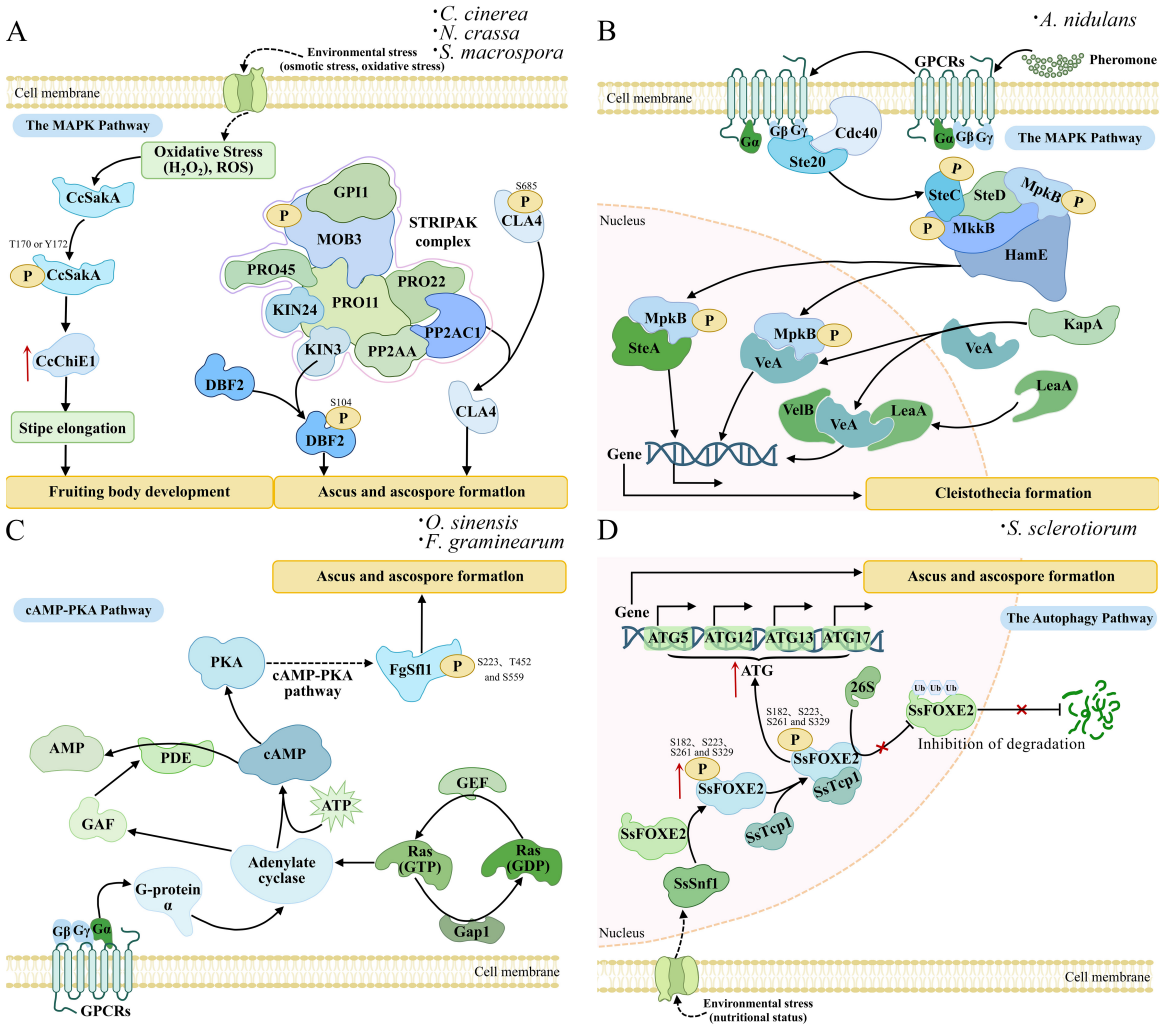
PTMs modulate sexual development in model filamentous fungi. **(A)** Under environmental stresses like osmotic or oxidative stress, membrane receptors activate the oxidative stress response pathway, leading to phosphorylation of the MAP kinase CcSakA at residues T170 and Y172. The Phosphorylated CcSakA upregulates the expression of CcChiE1, promoting stipe elongation and then fruiting body development. Besides, the STRIPAK complex regulates ascus and ascospore formation through the dephosphorylation of CLA4 at S685 by the PP2AC1 subunit and the phosphorylation of DBF2 at S104 by the KIN3 subunit, respectively. **(B)** The MAPK pathway initiates when external pheromones bind GPCRs, causing Gβγ dissociation and activating the SteC-MkkB-MpkB-SteD cascade via Cdc42-Ste20, with HamE facilitating MpkB phosphorylation. Phosphorylated MpkB then translocates to the nucleus to regulate transcription factors SteA and VeA, while KapA mediates VeA nuclear import for VelB/VeA/LaeA complex formation, collectively controlling cleistothecia development. **(C)** The cAMP-PKA pathway activates ascus and ascospores formation. The Gα subunit activates adenylate cyclase, with Ras-GTPase Ran to synthesize cAMP, while inhibiting GAF to prevent cAMP degradation. cAMP levels are elevated to activate PKA kinase, which phosphorylates FgSfl1 at S223/T452/S559 to regulate ascus and ascospore formation. Besides, the VelB/VeA/LaeA complex and PKA-mediated phosphorylation of FgSfl1 stage-specifically regulate gene expression for sexual reproduction. **(D)** Autophagy process regulates ascus and ascospore formation in *S. sclerotiorum* by integrating environmental stress signals. Under nutrient-limited conditions, membrane receptors perceive stress signals and relay them to the nucleus, where the SsSnf1 kinase phosphorylates SsFOXE2 at S182, S223, S261, and S329. Phosphorylated SsFOXE2 interacts with SsTcp1, and the complex binds to promoters of core autophagy genes, including *ATG5, ATG12, ATG13*, and *ATG17*, activating their transcription to initiate ascus differentiation and ascospore development.

The high-osmolarity glycerol mitogen-activated protein kinase (HOG) pathway are evolutionarily conserved signaling modules responding to environmental signals in eukaryotic organisms. In *Coprinopsis cinerea*, phosphoregulation of CcSakA modulates chitinase CcChiE1 expression and stipe elongation, linking oxidative stress responses to fruiting body morphogenesis (Figure 2A) (Zhao et al., 2022). Similarly, in *F. graminearum*, all three MAPKs fail to form perithecia, underscoring their essential role in sexual reproduction (Kong et al., 2018). Conserved MAPK-mediated signaling components, including Fus3, Hog1, Ste, and VE-1, fine-tune fungal sexual development through PTMs of their downstream targets. In *S. cerevisiae*, the mating pheromone responsive Fus3-MAPK cascade is activated via G-protein coupled receptors (GPCRs) and regulates mating gene transcription, while in *A. nidulans*, the pheromone module (SteC-MkkB-MpkB-SteD-HamE) controls cleistothecia formation through MpkB phosphorylation and its subsequent nuclear translocation, in which it interacts with TFs such as SteA and VeA (Frawley et al., 2018). Nuclear import of VeA is facilitated by KapA (α-importin) receptor binding to its NLS, enabling assembly of the VelB–VeA–LaeA velvet complex, governing the developmental gene expression during cleistothecia maturation (Figure 2B) (Frawley et al., 2018; Bayram et al., 2008). These findings highlight how MAPK cascades integrate with velvet complex, coordinating fungal sexual development in sexual reproduction in *N. crassa*.

MAPK cascades are pivotal regulators of virulence in prevalent phytopathogenic fungi. Key genes such as PMK1 MAPK in *M. oryzae* (Liu W. et al., 2021), Cmk1 in *Colletotrichum orbicular* (Harata and Kubo, 2014), Kpp2 (Ubc3) in *Ustilago maydis* (Kijpornyongpan and Aime, 2020), and Botrytis MAPK required for pathogenesis (*Bmp1*) in *Botrytis cinerea* (Jiang et al., 2018) have been frequently annotated as virulence factors. The Fus3/Kss1 homolog is the most extensively studied in fungal pathogens. The Fus3/Kss1 homolog PMK1 in *M. oryzae* regulates appressorium formation, invasive growth, and effector genes expression (Chen Y. et al., 2014). Its upstream kinase Ste7 acts as a pleiotropic regulator of morphogenesis and pathogenicity. In *Colletotrichum higginsianum*, ChSte7 is involved in regulation of vegetative growth, appressorial formation, and postinvasive growth in host tissues (Yuan et al., 2016). In *C. higginsianum*, ChSte7 controls vegetative growth, appressorium development, and postinvasive expansion in host tissues (Yuan et al., 2016). MAPK Crk1, a homolog of *S. cerevisiae* Ime2, is conserved among plant pathogenic fungi and regulates key developmental processes such as mating in *U. maydis* (Garrido et al., 2004).

Slt2 (Mpk1) cell integrity pathway monitors cell wall integrity (Xu et al., 1998). Several orthologs of yeast *SLT2* in plant pathogens have been characterized (Xu et al., 1998). This MAPK pathway coordinate cell wall biosynthesis and actin organization, supporting the development of penetration structures such as appressoria. Functional characterization of the *SLT2* homolog in several other plant pathogenic fungi. The *MPS1* MAPK is essential for conidiation, appressorial penetration, and infection in *Magnaporthe grisea* (Shi et al., 2024). Similarly, Slt2 homologs, such as MPS1 in *M. grisea*, coordinate cell wall integrity, actin organization, and penetration structure formation (Hou et al., 2002). In *F. graminearum*, the *mgv1* mutant is reduced in deoxynivalenol accumulation and hypersensive to plant defensin MsDef1 (Hou et al., 2002). Mgv1 also is essential for hyphal fusion and heterokaryon formation (Hou et al., 2002).

Hog1 homologs mediate cellular adaptation to osmotic stress, and play multiple roles in gene expression, cell cycle, and metabolic regulation, including the synthesis and retention of the compatible osmolyte glycerol to maintain cellular homeostasis under stress condition. For instance, in *Bipolaris oryzae*, deletion mutants of the HOG1 homolog *srm1* exhibited reduced growth under hyperosmotic conditions, in response to hydrogen peroxide (Moriwaki et al., 2006). In *B. cinerea*, the Hog1 homolog BcSak1 specifically regulates macroconidiation (Segmüller et al., 2007). The *bcsak1* mutants impair appressorium development and host penetration (Segmüller et al., 2007). Besides, HOG-MAPK inhibitors suppress nuclear localization of BcSAK1, spore germination and mycelial growth of *B. cinerea* at low temperature (Yan et al., 2025). So cold activates the HOG-MAPK pathway to regulate cold tolerance in this pathogen. Similar analyses of Hog1 homolog deletion mutants in *Fusarium asiaticum* and *Alternaria alternata* demonstrate that the HOG-MAPK pathway plays a highly conserved role in regulating the cold tolerance of postharvest pathogens and inactivation of this pathway effectively suppresses the development of cold-tolerant fungal diseases (Yan et al., 2025). Additionally, the scaffold protein Rack1 coordinates multiple processes, including vegetative growth, conidiation, mating, toxin biosynthesis, and stress responses, in various fungi through pathways such as cAMP/PKA and MAPK signaling (Zhang et al., 2016). Together, in plant pathogens, MAPK cascades, including Fus3/Kss1 MAPKs, Pkc1-Slt2 (Mpk1) cell integrity pathway, HOG-MAPK pathways, play important roles in virulence, probably for the adaptation to environmental cues and host immune responses. The core components of this pathway may be the potential targets for developing new fungicides.

#### 3.1.2 STRIPAK complex

The STRIPAK complex is an evolutionarily conserved supramolecular assembly that plays a critical role in protein (de)phosphorylation, thereby regulating cellular processes such as signal transduction and development. In fungi, this complex comprises multiple conserved subunits, including PRO11 (STRN), PRO22 (STRIP1/2), the structural and catalytic subunits of protein phosphatase 2A (PP2Aa and PP2Ac), Ham2, Ham3, Ham4, and monopolar spindle-one-binder 3 (Mob3) (Dettmann et al., 2013). Functionally, STRIPAK interacts with MAPK cascades. In *S. macrospora*, the subunits PRO11 and SmMOB3 interact to regulate cell fusion and the development of perithecia and ascospores (Bernhards and Pöggeler, 2011). A more detailed regulatory mechanism has been elucidated in *N. crassa*, where the terminal kinase of the MAPK cascade (MAK-2) phosphorylates the conserved N-terminal domain of MOB3 stabilizes STRIPAK assembly at the nuclear envelope and promotes nuclear accumulation of MAK-1 in CWI pathway (Figure 2A) (Dettmann et al., 2013; Fischer et al., 2018; Wilson et al., 2021). The STRIPAK complex coordinates ROS and osmotic stress responses to modulate sexual reproduction via PP2A-mediated dephosphorylation of stress-related MAP kinases, such as Hog1 and stress-activated kinase A (SakA). Beyond MAPK crosstalk, the PP2Aa mediates the dephosphorylation of the p21-activated kinase CLA4 at S685, which is essential for proper ascus and ascospore formation (Figure 2A) (Märker et al., 2020). The STRIPAK integrates with the target of rapamycin (TOR) pathway viaTap42-PP2A axis. Besides, the STRIPAK is genetically and physically associated with the Hippo-related septation initiation network (SIN) to regulate the nuclear Dbf2-related NDR kinase DBF2 for cell division during sexual spore formation (Shariatnasery et al., 2024). Besides, STRIPAK controls sexual development in *S. macrospora* through via differential phosphorylation of the RNA-binding protein Gul1, which shuttles dynamically along endosomes (Stein et al., 2020). Together, current research has substantially advanced the understanding of STRIPAK composition, assembly, and localization. However, the topological structure and dynamic regulation of the complex remain to be elucidated.

#### 3.1.3 Cyclic AMP-dependent protein kinase A

The cAMP-PKA pathway serves as a central signaling hub in fungi, regulating diverse developmental and pathogenic processes in fungi. In *Ophiocordyceps sinensis*, the cAMP pathway regulates the fruiting body development through a dual regulatory mechanism (Feng et al., 2017). Upregulation of G-protein β-subunit-like protein, Ras-GTPase Ran, and Rab GDP-dissociation inhibitor (RabGDI) boosts cAMP synthesis, while the downregulation of cGMP-specific phosphodiesterases, adenylyl cyclases, and FhlA (GAF) domain protein inhibits its degradation (Feng et al., 2017). This dual mechanism increases cAMP levels, leading to PKA activation to regulate sexual development (Figure 2C) (Feng et al., 2017). Similarly, in *L. edodes*, a putative G-protein γ-subunit gene (pri30080) is specifically expressed during primordia formation, indicating its role in developmental transition (Miyazaki et al., 2005). In *Schizophyllum commune*, chemical cues such as indole and caffeine promote fruiting body formation through increasing intracellular cAMP levels directly or via cAMP-degrading enzyme phosphodiesterase (PDE) inhibition (Kinoshita et al., 2002). However, the precise molecular targets and regulatory mechanisms by which these two chemical molecules modulate the cAMP signaling pathway remain to be further investigated (Kinoshita et al., 2002; Palmer and Horton, 2006).

In *F. graminearum*, the cAMP-PKA pathway plays a critical role in sexual reproduction and virulence. The transcription factor FgSfl1, a downstream target of this pathway, regulates perithecium formation and ascospore discharge through phosphorylation at multiple sites (S223, T452, S559) (Figure 2C) (Gong et al., 2021). In *F. graminearum*, two genes *cpk1/2*, encoding the catalytic subunits of cAMP- PKA, are required for perithecia development and pathogenicity (Hu et al., 2014). Besides, the deletion of *GIA1*, a non-pheromone GPCR, results in the formation of normal perithecia but disrupts meiosis in *F. graminearum* (Ding et al., 2023). Besides, G-protein-coupled receptors (GPCRs) such as the non-pheromone receptor Gic1-Interacting Protein 1 (Gip1) coordinate early sexual development by activating both cAMP and Gpmk1 pathways, potentially through downstream regulators like FgVeA (Ding et al., 2025). Notably, in *F. graminearum*, ~61% of Gip1transcripts undergo A-to-I mRNA editing, resulting in the S448G missense mutation in the C-terminal region that affects its intracellular signaling (Liu et al., 2016). PM of RNA editing of GPCR receptors further expand the regulatory dimensions of this pathway. However, the mechanisms underlying upstream receptor recognition, the identity of chemical molecules, and the dynamic regulatory network, require in-depth investigation. Additionally, the cAMP-PKA signaling pathway is essential for infection-related morphogenesis in fungal pathogens, notably driving appressorium differentiation in *M. oryzae*. E.g., the Suppressor of Mitotic instability 1 (MoSom1) protein, functioning downstream of cAMP-PKA pathway, is essential for infection-related morphogenesis and pathogenicity in *M. oryzae* (Yan et al., 2011). Notably, serine 227 in MoSom1 has been identified as a putative PKA phosphorylation site, and its phosphorylation is critical for regulating these infection-related processes (Yan et al., 2011).

#### 3.1.4 Transcription

TFs involved in sexual reproduction in filamentous fungi have been characterized. Genome-wide expression profiling in *S. commune* identified 283 and 253 TFs specifically expressed during primordium formation and fruiting body development, respectively (Ohm et al., 2010). Five TFs genes of *S. commune*, including *hom1, hom2, c2h2, gat1*, and *briI*, are involved in fruiting body formation (Ohm et al., 2010). Similarly, in *Podospora anserina*, five HMG and two homeodomain (hom) TFs are required for the development of the fruiting body (Ait Benkhali et al., 2013). The *hom2* knockout strains displayed 30% faster mycelial growth but failed to form a fruiting body (Ohm et al., 2011). Furthermore, phosphoregulation of Hom2 further fine-tunes this developmental switch: dephosphorylation of its four predicted RRXS motifs slows mycelial growth yet accelerates fruiting-body initiation, demonstrating that phosphorylation-dependent modulation of Hom2 controls the transition from vegetative growth to sexual reproduction (Pelkmans et al., 2017).

White collar-1 (WC-1) interacts with White collar-2 (WC-2) to form White Collar Complex (WCC), regulating transcription of genes required for early sexual development and circadian rhythms (Kim et al., 2015). In *Neurospora*, WC-1 functions as the primary blue-light photoreceptor and a core positive element in the circadian feedback loop under constant darkness, primarily by activating transcription of the frequency (frq) gene (He et al., 2005). Tandem mass spectrometry has identified five *in vivo* phosphorylation sites clustered downstream of the WC-1 zinc-finger DNA-binding domain (He et al., 2005). PM at these sites negatively regulates WC-1′s function within the circadian feedback loop and is essential for the function of the *Neurospora* circadian (He et al., 2005). Light-induced phosphorylation of WC-1 and WC-2 occurs exclusively in the nucleus, suggesting that this PM may regulate their nuclear localization or DNA-binding activity (Schwerdtfeger and Linden, 2000). Moreover, PKC can phosphorylate the WC-1 zinc finger region *in vitro*, this specific activity is unlikely to mediate PKC's function in regulating light responses *in vivo* (Franchi et al., 2005). Notably, proline residues are adjacent to two phosphorylation sites, suggesting that phosphorylation might be regulated by a proline-directed kinase, a glycogen synthase kinase-3 (GSK-3), a known regulator of the circadian clock in *Drosophila* (Ko et al., 2010). These findings contribute to a deeper understanding of the mechanisms by which TFs regulate sexual development, however how these TFs synergize with PM regulatory networks resulting in sexual development remains elusive.

#### 3.1.5 Autophagy

Autophagy is a common, evolutionarily conserved decomposition cycle pathway in eukaryotic cells under stress and helps to maintain protein homeostasis in cells. Autophagy also plays a role in cell differentiation and pathogenesis of pathogenic fungi. For instance, in pathogenic fungi such as *M. oryzae* and *F. graminearum*, autophagy is involved in infection-related processes, including germination, appressorium formation, and glycogen utilization (Miguel-Rojas et al., 2023; Josefsen et al., 2012; Nguyen et al., 2011). In *S. sclerotiorum*, autophagy-related genes 8 (Atg8) interacts with the cargo receptor SsNBR1 and the TF FORKHEAD BOX E3 (SsFoxE3) to regulate sclerotia formation, appressoria development, and pathogenicity (Jiao et al., 2022). Moreover, phosphorylated SsFoxE2 interacts with translationally-controlled tumor protein 1 (SsTctp1) and binds to promoters of autophagy-related (ATG) genes and induce their transcription (Figure 2D) (Zhu et al., 2025). SsFoxE2- regulated autophagy influences the ubiquitination balance and early fruiting body development, thereby directly influence the sexual development and virulence (Zhu et al., 2025).

#### 3.1.6 Pathogen infection

In the long-term evolutionary battle with plants, pathogenic fungi have developed sophisticated strategies to manipulate host immune system by delivering effector proteins into plant cells. Targeting protein PM has emerged as a central mechanism of attack. Many effectors act as kinase inhibitors or phosphatases to directly suppress the activity of defense-related proteins. For instance, *Phytophthora infestans* effector PexRD2 physically interacts the phosphorylation site of MAPKKKε, disrupting the MAPK signaling cascade (Ren et al., 2019). Some bacteria effectors function as active kinases to aberrantly phosphorylate host proteins. Examples include the *Xanthomonas euvesicatoria* effector XopAU, which phosphorylates and activates MKK2 to perturb immune signaling (Teper et al., 2018), and the *Pseudomonas syringae* effector HopBF1, which phosphorylates and inhibits the chaperone HSP90, thereby preventing proper folding of immune receptors (Lopez et al., 2019). Additionally, *P. syringae* effectors such as AvrB can hijack host kinases like RIPK to modify intermediary proteins like RIN4, indirectly suppressing Pathogen-Associated Molecular Patterns (PAMPs)-triggered immunity (PTI) while potentially triggering Effector-Triggered Immunity (ETI) (Liu et al., 2011). These diverse tactics highlight how pathogens have evolved to precisely rewire the host's phospho-regulatory network, effectively dismantling layered plant defenses.

Collectively, PM plays a central role in the integration of signaling pathway, transcription regulation, stress response, fundamental cellular process such as autophagy, thereby orchestrating fungal sexual development, environmental adaptation, and virulence, yet the molecular mechanisms underlying crosstalk between these pathways and how the environmental cues are integrated into these phosphoregulatory networks remain to be fully elucidated.

### 3.2 Acetylation modification

AM refers to the enzymatic transfer of an acetyl group from acetyl-CoA to the lysine residues of proteins, forming acetylated lysine. Lys AM on histones H3 and H4 is the most extensively studied AM event and plays a fundamental role in chromatin regulation. Histone AM is regulated by histone acetyltransferases (HATs) and histone deacetylases (HDACs). HATs are classified into nuclear type A and cytoplasmic type B based on their subcellular localization (Kong et al., 2018). HDACs are classified into three families: sirtuins, classical HDACs, and HD2-like enzymes (Brosch et al., 2008). Nuclear histone AM reduces chromatin compaction to promote gene transcription. During host infection, pathogenic fungi convert chitin to chitosan through deacetylation, protecting their hyphae from host chitinase recognition and avoiding chitin-triggered immunity.

**Figure 3:**
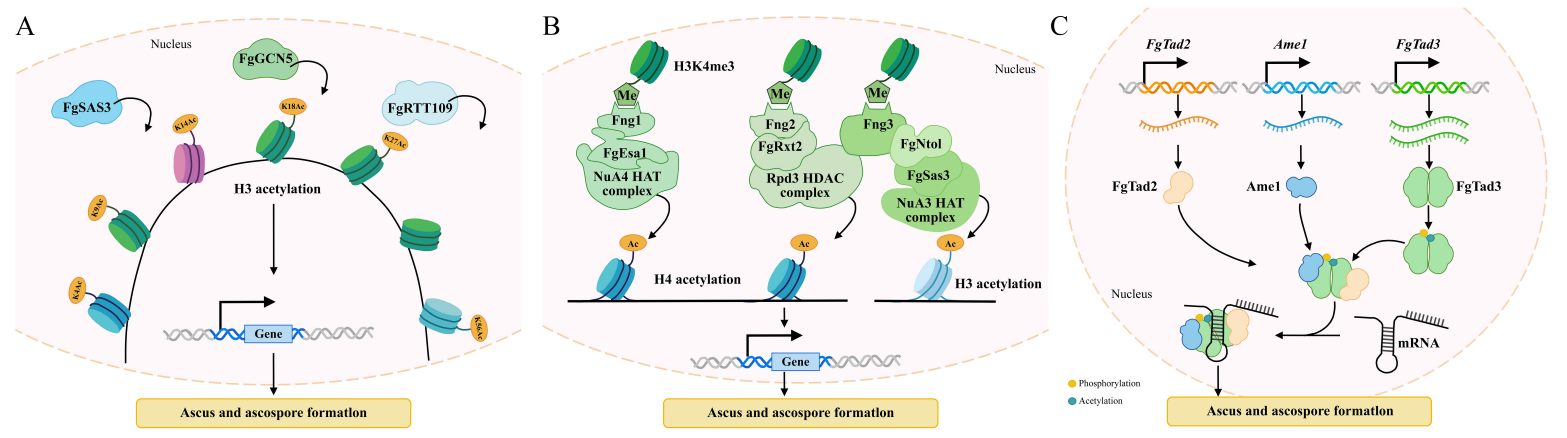
AM modulates sexual development in filamentous fungi such as Fusarium spp. **(A)** AM regulates the formation of ascus and ascospore formation. HATs (FgGCN5, FgSAS3, and FgRTT109) coordinately regulate sexual development through site-specific histone H3 modifications. In the nucleus, FgGCN5 acetylates H3 acetylation at K9/K14/K18/K27, FgSAS3 acetylates H3 at K4/K14, H3K56 acetylation is exclusively regulated by FgRTT109. These modifications induce chromatin relaxation and activate transcription for ascus differentiation and ascospore formation. **(B)** ING Family Proteins Integrate Histone Modification Complexes in Sexual Development. Fng1 recruits the NuA4 HAT complex via FgEsa1 to catalyze H4 acetylation at K5, K8, K12, and K16, activating sexual reproductive gene expression. Fng2 associates with the FgRxt2-containing Rpd3 HDAC complex to mediate H3/H4 deacetylation. Fng3 simultaneously engages the NuA3 HAT complex for H3 acetylation (K4, K14, K36) and the FgRpd3 HDAC complex for H4 deacetylation (K8, K12, K16). These coordinated activities, triggered by H3K4me3 recognition, fine-tune gene expression during ascus development and ascospore maturation. **(C)** A-to-I mRNA Editing in Sexual Reproduction Regulation. During sexual reproduction, FgTad2, Ame1, and FgTad3 assemble into an RNA editing complex. The CDA domains of FgTad2 and FgTad3 form the catalytic core, while Ame1 binds the N-terminal domain of FgTad3 to recognize mRNA substrates. The complex is stabilized and its editing efficiency enhanced by PTMs of FgTad3, including acetylation at K198 and phosphorylation at E241. The assembled complex conducts A-to-I editing on target transcripts, precisely regulating genes involved in ascus morphogenesis and ascospore maturation.

HATs are central epigenetic regulators that critically influence fungal virulence, stress adaptation, and secondary metabolism. In *F. graminearum*, putative HATs (FgGCN5, FgSAS3, FgRTT109) mediate specific AM on histones H3 and H4, with the deletion mutants showing impaired perithecium formation and ascospore production (Figure 3A) (Kong et al., 2018). The ING protein family members (Fng1-3) serve as critical adaptors, with Fng1 binding the NuA4 HAT complex subunit FgEsa1 to regulate H4 AM and perithecium initiation, Fng3 interacting with the NuA3 HAT complex (via FgSas3 and FgNto1) to promote H3 AM and the Rpd3 HDAC complex to deacetylate H4, maintaining an acetylation-deacetylation equilibrium required for asci and ascospore maturation, and Fng2 associated with the Rpd3 HDAC complex to modulate H3/H4 deacetylation, hyphal growth, virulence, and perithecium development (Figure 3B) (Jiang et al., 2020). MYST family is the largest group of HATs, such as Sas3, which play critical and conserved roles in regulating fungal development and pathogenicity across diverse species. In *A. fumigatus*, Sas3 localizes to the nucleus, acetylates histone H3 at lysine 9 and 14 (H3K9/K14). It is essential for colony growth, conidiation, virulence, and cell wall integrity. This is observed hypersensitivity of *Sas3* deletion mutants to cell wall-perturbing agents, the altered cell wall thickness, and the dysregulated phosphorylation of the cell wall integrity (CWI) kinase MpkA (Wang et al., 2024). Site-directed mutagenesis further revealed that residues G641, G643, and E664 are collectively required for Sas3′s acetylation, with only the triple mutant phenocopying the Δ*sas3* defects, suggesting Sas3 may functions beyond histone AM (Wang et al., 2024). Similarly, in *M. oryzae*, loss of MoSAS3 severely impairs pre-penetration development and pathogenicity (Dubey et al., 2019). These findings underscore Sas3 and its orthologs act as multifunctional epigenetic regulators and are potential targets for controlling pathogenic fungi (Dubey et al., 2019). RTT109 is a unique histone acetyltransferase specifically acetylating histone H3 lysine 56 (H3K56), a modification vital for DNA replication-coupled nucleosome assembly and genome stability (Zhang X. et al., 2024). In pathogenic fungi such as *A. flavus, A. fumigatus*, and *Monascus* spp., RTT109 orchestrates conidiation, secondary metabolism and virulence (Zhang X. et al., 2024). Structural studies of the Rtt109-Asf1-H3-H4 complex reveal that unwinding of the histone H3 αN-helix (where K56 resides) and stabilization of the C-terminal β-strand of H4 by Asf1 are prerequisites for H3K56 acetylation (Zhang et al., 2018). An interaction between Rtt109 and the central helix of histone H3 is also required for its AM function (Zhang et al., 2018). The structural characterization of the H3K56 HATs is anticipated to develop targeted agents against fungal infections (Zhang et al., 2018). The insight into a histone-modifying enzyme engaged with a multiprotein substrate unveils the intricate molecular interactions underlying substrate specificity (Zhang et al., 2018), Elongator Protein 3 (Elp3), the catalytic subunit of the elongator complex, belongs to the Gcn5-related N-acetyltransferase (GNAT) family of HAT and serves as a central epigenetic mechanism governing fungal sexual development and morphogenesis (Winkler et al., 2002). Elp3 facilitates chromatin opening and gene activation by acetylating N-terminal lysine residues on histones H3 and H4 (Winkler et al., 2002; Mengke et al., 2022). In *F. graminearum*, deletion of *ELP3* reduces H3K14 acetylation levels, leading to enhanced chromatin condensation and downregulation of genes-associated with perithecium development and sporulation (Lee et al., 2014). This disruption leads to reduced perithecium production, delayed perithecium maturation, and abnormal ascospore development (Lee et al., 2014).

HDACs play multifaceted and essential for regulating growth, development, and virulence across a wide range of filamentous fungi. In *B. bassiana*, RPD3/HDAC1, a class I histone deacetylase, reverses lysine acetylation, mediates growth, asexual development and virulence (Zhang Y. et al., 2024). Deletion of Rpd3 results in widespread hyper-or hypoacetylation across lysine residues of all four core histones (H2A, H2B, H3, H4) and multiple histone acetyltransferases, indicating that Rpd3 directly or indirectly genome-wide lysine modification landscapes (Zhang Y. et al., 2024). Similarly, in pathogens like *M. oryzae* and *B. cinerea*, RPD3 overexpression can disrupt infection structure development, oxidative stress tolerance, and pathogenicity (Zhang X. et al., 2024). Moreover, in *C. heterostrophus*, two components of the Rpd3 HDAC complex, ChPho23 and ChSds3, were identified as being involved in the nitrosative stress response and virulence. ChPho23 and ChSds3 directly interact with ChHog1, which in turn associates with ChCrz1 to up-regulate the transcription of genes involved in the nitrosative stress response, thereby enabling *C. heterostrophus* to cope with nitrosative stress (Fan et al., 2025). These findings provide a potential foundation for developing targeted control strategies against sclerotinia leaf blight (SCLB) that target ChPho23 and ChSds3 (Fan et al., 2025). HOS2, a Class II HDAC, is also crucial for virulence, influencing extracellular depolymerase expression in *C. carbonum* (Zhang X. et al., 2024). In *U. maydis*, Hos2 acts as a downstream component of the cAMP-PKA pathway, regulating the expression of MAT genes through deacetylating histone H4 at lysine 16 (H4K16) (Zhang X. et al., 2024). Clr3, another HDAC in *U. maydis*, also participates in the cAMP-dependent transcriptional control of MAT genes (Zhang X. et al., 2024). These findings provide new insights into the role HDACs in fungal development and pathogenesis. Beyond histones, HDACs like RPD3 and HDA1 can target nonhistone proteins, such as HSP90 or autophagy factors, linking acetylation directly to cellular adaptation and pathogenicity. Sirtuins, Class III HDACs, are integral to chromatin silencing and virulence. In *M. oryzae*, SIR2 contributes to the infection by deacetylating the Jumonji C domain-containing histone demethylase (MoJMJC) repressor, leading to the upregulation of superoxide dismutase (SOD) expression (Fernandez et al., 2014). Consequently, enhanced ROS detoxification promotes pathogen infection (Fernandez et al., 2014). Besides, Proteomic profiling during sclerotium formation in *Polyporus umbellatus* identified numerous acetylated proteins enriched in ROS metabolic pathways (Li B. et al., 2022). Functional validation confirmed that oxidative stress promotes sclerotium formation via protein AM, revealing an interplay between ROS signaling and AM in regulating sexual development. Future studies should further elucidate how environmental cues like oxidative stress are integrated with AM to orchestrate complex sexual developmental in fungi.

### 3.3 Methylation modification

**Figure 4:**
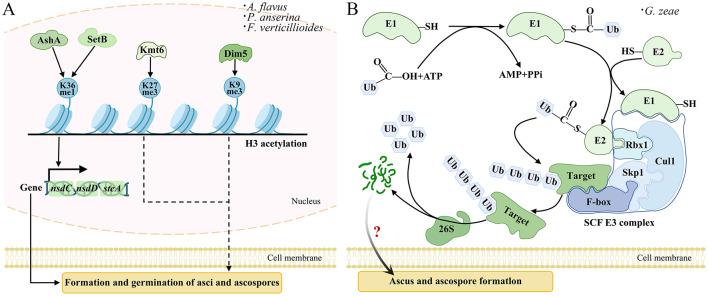
MM and UM modulates sexual development in filamentous fungi. **(A)** Two key methyltransferases, AshA and SetB, promote the expression of developmental regulators (nsdC, nsdD) and the sexual reproduction gene steA by H3K36 methylation, initiating sexual development. Kmt6 catalyzes H3K27me3, while Dim5 mediates H3K9me3, precisely controlling the spatiotemporal expression of genes involved in ascus formation and ascospore germination. **(B)** Ubiquitin is activated by the E1 in an ATP-dependent manner, forming a thioester bond with E1. The activated ubiquitin is then transferred to an E2 conjugating enzyme and delivered to target proteins that are recognized by E3 ligases through ubiquitin. The target protein is recognized by the E3 ligase and labeled with ubiquitin. This process is recycled to produce polyubiquitin chains that label the proteins for degradation by the 26S proteasome, dynamically controlling protein levels related to ascus and ascospore formation.

In fungi, histone lysine methylation represents the predominant form of histone modification, catalyzed by histone lysine methyltransferases (HKMTs). These HKMTs typically contain the catalytic SET domain (Su (var) 3-9, enhancer of zeste, trithorax domain) (Zhang and Tao, 2025), transferring methyl groups from S-adenosylmethionine (SAM) to N-terminal lysine residues of histone H3 or H4 (Lai et al., 2022). In *Fusarium verticillioides*, the H3 lysine 9 (H3K9) methyltransferase FvDim5 regulates H3K9me3 trimethylation, regulating perithecium formation and sexual development (Figure 4A) (Gu et al., 2017). Similarly, in *P. anserina*, the EZH2-like protein PaKmt6 acts as the core catalytic subunit of polycomb repressive complex 2 (PRC2), catalyzing H3 lysine 27 trimethylation (H3K27me3) to control gene silencing (Figure 4A) (Carlier et al., 2021). Disruption of PaKmt6 abolishes H3K27me3, resulting in transcriptional dysregulation, defective fruiting body morphogenesis, severely impaired spore germination, and aberrant overproduction of male gametes (Figure 4A) (Carlier et al., 2021). In *A. flavus*, the Set2 family methyltransferases AshA and SetB regulate H3 lysine 36 (H3K36) methylation, modulating the expression of sclerotia-regulatory genes *nsdC* and *nsdD*, as well as the key sexual development gene *steA*, influencing sclerotia formation and sexual reproduction (Figure 4A) (Zhuang et al., 2022). In *Aspergillus spp*., overexpression of *SasA* resulted in few, underdeveloped, and sterile cleistothecia that lack fertile ascospores (Gerke et al., 2012). Genetic and biochemical evidence suggest that SasA physically interacts with LaeA, a putative methyltransferase containing conserved SAM-binding domains, which is critical for normal fruiting body maturation (Gerke, 2012). Dysregulation of the SasA-LaeA complex disrupts ascospore production and sexual development. These studies demonstrate that MM serves as a key regulatory mechanism governing the fruiting body morphogenesis, and ascospore germination and formation in fungi.

### 3.4 Ubiquitination modification

Ubiquitination is a 76aa protein attached to target Lys residues through sequential enzymatic actions involving an activating enzyme (E1), a conjugating enzyme (E2), and a ligase (E3). This process can result in either monoubiquitination, which often alters protein localization or activity, or polyubiquitination, typically linked to protein degradation (Dikic and Schulman, 2023; Seymour et al., 2016; Fu et al., 2018; Xiang et al., 2015). Polyubiquitin chains are formed through specific linkages, including Lys6 (K6), Lys11 (K11), Lys27 (K27), Lys29 (K29), Lys33 (K33), Lys48 (K48), Lys63 (K63), or linear (M1) linkages, each associated with distinct functional outcomes (Huang et al., 2016). The Skp1-Cullin (Cul1)-F-box protein (SCF) represents a well-studied RING-type E3 (Figure 4B) (Cardozo and Pagano, 2004), where F-box proteins serve as essential SCF components (Liu and Xue, 2011).

In *Gibberella zeae*, FBP1, a multifunctional F-box protein, functions by assembling into SCF complexes that ubiquitinate key developmental regulators (Figure 4B) (Han et al., 2007), highlighting the central role of SCF-mediated ubiquitination in fungal development. Comparative transcriptomic analysis across six fungal species revealed that genes encoding F-box proteins, RING-type zinc-finger proteins, and BTB/POZ domain proteins were highly upregulated during the primordium stage, suggesting that fruiting body development may be critically regulated by RING-type E3-mediated UM (Krizsán et al., 2019). Deletion of the polyubiquitin-encoding gene *UBI4* impairs hyphal growth, sporulation, germination, appressorium formation, and pathogenicity (Oh et al., 2012). Key ubiquitination components identified in *M. oryzae* include the E2 enzyme Rad6, which collaborates with at least three E3 ligases to degrade targets involved in degradation, histone regulation, DNA replication, and SCF complex activity (Liu W. et al., 2021). The scaffold protein MoSkp1 assembles with 17 F-box proteins to form functional SCF complexes, among which at least three are essential for virulence (Baudin et al., 2024). Notably, the F-box protein MoGrr1, a homolog of regulators also important in *Fusarium oxysporum*, is critical for conidiogenesis and pathogenicity, illustrating the conserved role of SCF-F-box modules in fungal infection processes (Tan et al., 2023).

## 4 Novel PTMs

Except for above conventional PTMs, some novel PTMs were implicated in the fungal morphology, hyphal development, differentiation, and fungal virulence, including protein O-glycosylation (GlcNAc), Ksucc and SUMOylation. For instances, Ksucc is implicated in fungal virulence, positions it as a promising therapeutic target for novel antifungal strategies (Sreedhar et al., 2020). SUMOylation components are primarily localized in the nucleus and are highly expressed during macroconidia germination (Azizullah et al., 2023). Knockout of these genes led to significant defects in vegetative growth, asexual reproduction, conidial morphology, and spore germination (Azizullah et al., 2023; Chen et al., 2020; Mei et al., 2023; Harting et al., 2013; Lara-Ortíz et al., 2003; Wang et al., 2023; Lee and Shaw, 2008; Duan et al., 2024).

### 4.1 Glycosylation

Glycosylation is a key PTM in which sugar chains are covalently attached to specific amino acid residues of proteins, forming glycoproteins. It is primarily classified into two types: N-land O-glycosylation. N-glycosylation occurs on the asparagine residue within the conserved sequence Asn-X-Ser/Thr (X is any amino acid except proline) and is initiated in the endoplasmic reticulum (ER) (Kelleher and Gilmore, 2006). This modification enhances protein stability and plays a vital role in protein trafficking, localization, cell recognition, and signal transduction (Liu C. et al., 2021).

In plant pathogenic fungi, a total of 559 N-glycosylation sites across 355 proteins were identified and quantified at different developmental stages (Chen et al., 2020). Functional analysis revealed that N-glycosylation coordinates multiple cellular processes essential for mycelial growth, conidium formation, and appressorium development, with particularly high levels of modification observed during conidial and appressorium stages (Chen et al., 2020). Phenotypic screening of gene deletion mutants identified four endoplasmic reticulum (ER) quality control (ERQC) components (Gls1, Gls2, GTB1, and Cnx1) as critical for mycelial growth, conidiation, and invasive hyphal growth in host cells (Chen et al., 2020). Further investigation showed that the N497 glycosylation of glutamine synthetase 1 (Gls1) is essential for invasive hyphal growth and partially required for conidiation (Chen et al., 2020). Mutation of N497 led to reduced Gls1 protein levels and mislocalization from the ER to the vacuole, indicating its importance for Gls1 stability (Goto, 2007). N-glycosylation-deficient mutants in *U. maydis* (Marín-Menguiano et al., 2019), *M. oryzae* (Chen X. L. et al., 2014), and *Mycosphaerella graminicola* (Liu W. et al., 2021) have demonstrated the critical role of this modification in fungal plant pathogenesis. In *F. oxysporum*, deletion of the N-glycosyltransferase *Gnt2* alters protein glycosylation profiles, leading to defects in conidium morphology, hyphal fusion efficiency, and the secretion of transport vesicles and their protein cargo (López-Fernández et al., 2013). In *M. graminicola*, an α-mannosyltransferase regulates the transition from yeast-like to hyphal growth (Motteram et al., 2011), while in *M. oryzae*, a distinct mannosyltransferase is necessary for suppressing host ROS production (Chen X. L. et al., 2014). Inhibition of N-glycosylation in *Colletotrichum graminicola*, the causal agent of maize anthracnose, severely impaired vegetative growth, hyphal tipdevelopment, conidial germination, appressorium formation, and host infection capacity (Mei et al., 2023). Quantitative proteomics further indicated that N-glycosylation functionally coordinates with O-glycosylation, glycosylphosphatidylinositol (GPI) anchor modifications, and endoplasmic reticulum (ER) quality control (ERQC) by directly targeting proteins in these pathways (Mei et al., 2023). Functional studies of the N-glycosylation-related protein asparagine-linked glycosylation 3 (ALG3) and the ERQC-related protein calnexin 1 (CNX1) demonstrated that N-glycosylation of ER chaperones is essential for effector stability, secretion, and overall pathogenicity in *C. graminicola* (Mei et al., 2023).

*N*-glycosylation of effector proteins is a common strategy to help fungal pathogens evade host innate immunity. Key effector proteins, such as the chitin-binding effector Slp1 in *M. oryzae*, require N-glycosylation at multiple sites to suppress chitin-triggered immunity, with glycosylation enhancing both chitin-binding capacity and protein stability (Chen X. L. et al., 2014). Putative N-glycosylation sites are also found in effectors from other pathogens, including *U. maydis* Pep1 and Pit1 (Doehlemann et al., 2009; Fernández-Álvarez et al., 2012), *Cladosporium fulvum* Ecp6 (Sánchez-Vallet et al., 2013), and *M. oryzae* Bas4 (Mosquera et al., 2009). In *U. maydis*, the O-mannosyltransferase Pmt4 affects virulence through the glycosylation of effectors such as Pit1 and Um03749, both essential for biotrophic growth (Fernández-Álvarez et al., 2012).

O-glycosylation, particularly O-mannosylation, represents the most prevalent and well-characterized form of glycosylation in fungal plant pathogens, capable of incorporating a broader diversity of sugars compared to N-linked modifications. In filamentous fungi, O-glycan structures are highly host-dependent (Deshpande et al., 2008). Disruption of native O-glycans often leads to the loss of protein function, *in vivo* half-life, and immunogenicity (Wopereis et al., 2006). Studies have confirmed that O-glycosylation is essential for maintaining fungal morphology, hyphal development, and cellular differentiation (Goto, 2007). Due to its functional significance, glycoengineering, particularly remodeling N-glycan structures to produce humanized glycoproteins in yeast and filamentous fungi, has become a major focus of biotechnology.

### 4.2 SUMOylation

SUMOylation is an evolutionarily conserved PTM involving the covalent attachment of small ubiquitin-like modifier (SUMO) proteins to Lys residues of target proteins. Similar to UM, this process is catalyzed by a dedicated enzymatic cascade (E1 activating, E2 conjugating, and E3 ligase enzymes) and primarily regulates transcriptional activity, protein stability, and signal transduction (Zhao, 2007). SUMOylation has emerged as a central mechanism coordinating both virulence and development in fungal pathogens. For instance, in *M. oryzae*, knockout of the SUMOylation pathway genes [e.g., *SMT3* (SUMO), *AOS1* (E1), *UBA2* (E1), *UBC9* (E2), and *SIZ1* (E3)] impairs hyphal growth, sporulation, and conidiophore development, underscoring its essential role in pathogenesis (Liu et al., 2018). Moreover, numerous pathogenesis-related proteins, including key regulators of appressorium formation, ROS detoxification, and effector trafficking and secretion, undergo SUMOylation (Liu W. et al., 2021). Deletion of SUMOylation significantly impairs host penetration and invasion. Fungal pathogens also manipulate the host SUMOylation to suppress plant immunity. For instances, *Trichoderma viride* effector ethylene-inducing xylanase (EIX) reduces tomato SUMO transcript levels, leading to activation of defense response (Sharma et al., 2021), while *Plectosphaerella cucumerina* SUMO-activating enzyme subunit 2 (SAE2) and SUMO-activating enzyme subunit 1 (SCE1) effectors impairs SUMO conjugation by affecting E1 and E2 enzyme turnover (Castaño-Miquel et al., 2017). These findings collectively demonstrate that diverse pathogens converge on targeting SUMOylation as a key strategy to defense plant immunity and establish host colonization. SUMOylation also governs the proper localization of septins, which is essential for appressorial actin ring formation during infection. SUMO potentially interacts the velvet complex component RcoA to regulate sexual development (Harting, 2013). Moreover, SUMO integrates stress response pathways with sexual development, influencing oxidative stress through target proteins like SodA (a superoxide dismutase) and yeast Ntg1 homologs involved in oxidatively damaged DNA (Harting et al., 2013). The deletion of the NADPH oxidase gene *noxA* blocks sexual development (Lara-Ortíz et al., 2003). Collectively, these findings highlight SUMOylation as a multifaceted hub that synchronizes pathogenicity, stress adaptation, and developmental transitions in fungal pathogens.

### 4.3 Ksucc

Ksucc is an evolutionarily conserved and reversible post-translational modification that substantially alters the chemical and structural properties of target proteins by adding a succinyl group to a lysine residue (Zheng et al., 2025). The dynamics of Ksucc are governed by a balance between succinyltransferases and desuccinylases (Sreedhar et al., 2020). Fungal studies illuminate the potential role of Ksucc in regulating secondary metabolism. For instance, in *A. flavus*, a quantitative proteomic study identified widespread Ksucc, and follow-up experiments confirmed that Ksucc of a key enzyme, versicolorin B synthase, directly influences aflatoxin biosynthesis and sclerotia development (Wang et al., 2023). Notably, many enzymes in biosynthetic gene clusters are also acetylated at the same lysine residues, suggesting that these PTMs crosstalk may be an alternative regulatory mechanism for secondary metabolite production. In *G. lucidum*, 47 succinylated enzymes involved in the biosynthesis of triterpenoids and polysaccharides were identified (Wang et al., 2019). Recently, increased studies underscored the potential significance of Ksucc in various biological processes, encompassing normal physiological functions and the development of pathological processes and metabolites (Adejor et al., 2024). Global succinylome analyses showed that thousands of succinylation sites have been identified in several pathogenic fungi, including *Magnaporthe oryzae* (Ren et al., 2024), *Trichophyton rubrum* (Xu et al., 2017), and *A. flavus* (Ren et al., 2018). Ksucc is suggested to play a key role in core metabolic regulation in *P. oryzae*, and notably, more than 40 pathogenicity-related proteins in this fungus were found to be succinylated, linking this modification closely to its virulence. In *T. rubrum*, a common dermatophyte, succinylated proteins are involved in diverse cellular functions such as translation, epigenetic regulation, and metabolism. Moreover, 24 proteins associated with pathogenicity were also observed to be succinylated. Similarly, in *Candida albicans*, a major human fungal pathogen, one of the most prevalent human fungal pathogens, may rely on protein succinylation to critically regulate the TCA cycle. Thus, targeting succinylation (Ksuc) could represent a promising strategy for reducing fungal pathogenicity. In our study, comparative analysis of Ksucc between the sclerotium (ST) and primordium (PR) formation stages, the initial phase of sexual development in *O. sinensis*, revealed that, among 180 identified Ksucc sites across 86 proteins, all but one sites were significantly upregulated during the PR stage compared to the ST stage. This extensive upregulation strongly suggests that Ksucc plays a pivotal regulatory role in sexual morphogenesis in this species (data not shown).

### 4.4 Other novel PTMs

Protein lipidation refers to the process by which fatty acids or isoprenoid groups are covalently attached to proteins. The three common types of lipidation modifications are myristoylation, palmitoylation, and prenylation (Resh, 2012). Among these, myristoylation involves the attachment of myristic acid via an amide bond to the N-terminal glycine residue of a protein, which can affect the membrane localization and conformation of the protein. Disruption of ADP-ribosylation factor B (ArfB), a protein containing an N-myristoylation motif, leads to failure in the hyphal polarity during early morphogenesis and a delay in endocytosis in *A. nidulans* (Lee and Shaw, 2008; Bergs et al., 2016).

Protein S-acylation or S-palmitoylation is a PTM process mediated by the family of palmitoyl acyltransferases (PATs), which attach fatty acyl chains to cysteine residues via thioester bonds (Zhang and Hang, 2017). The modification facilitates membrane association, induces conformational changes, modulates protein stability and protein-protein interactions, and often acts with other lipid modifications like myristoylation and prenylation to fine-tune protein localization and function (Zhang and Hang, 2017; Rocks et al., 2005; Kanaani et al., 2008). In *Ustilaginoidea virens*, S-palmitoylation modification is involved in multiple signaling pathways, including MAPK, autophagy, and the proteasome system (Duan et al., 2024). S-palmitoylation by the palmitoyltransferase UvPfa4 enhances the virulence of *U. virens* through its modification of the MAP kinase UvSlt2 (Duan et al., 2024). This palmitoylation boosts the kinase's phosphorylation activity, which increases the hydrophobic solvent accessible surface area and strengthens the binding between UvSlt2 and its substrate, UvRlm1 (Duan et al., 2024). This finding S-palmitoylation plays a critical role in regulating pathogen virulence (Duan et al., 2024).

Neddylation refers to the addition of the NEDD8 polypeptide to lysine residues of a small range of target proteins. It is essential for fungal growth and closely linked to Um, regulating the cullin-1 protein of the SCF E3 ligase complex. A neddylation protein ortholog exists in *M. oryzae*, but the role of neddylation in plant pathogenesis remains largely unknown. The COP9 signalosome (CSN) is a conserved protein complex that regulates cullin-RING ubiquitin ligases (CRLs) by the removal of Nedd8 from cullin subunits (Braus et al., 2010; Nagy et al., 2023). In *A. nidulans*, the deletion of *csnD* and *csnE* (encoding the 4th and 5th subunits of CSN, respectively) allowed primordia formation but blocked the development of mature cleistothecia or fruiting bodies, suggesting that the CSN complex is crucial for UM-mediated sexual reproduction (Busch et al., 2003). However, the specific molecular targets of CSN-regulated CRLs remain to be fully elucidated (Busch et al., 2003).

Glycosylphosphatidylinositol (GPI) anchoring is a complex and fundamental PTM essential for the localization and function of extracellular proteins in eukaryotes. In plant pathogenic fungi such as *F. graminearum* (Rittenour and Harris, 2013), *A. fumigatus* (Samalova et al., 2020), and *M. oryzae* (Chen et al., 2020), GPI-anchored proteins are critical for virulence and immune evasion. These proteins play a key role in fungal cell wall biogenesis and integrity, largely through the PTM of structural mannoproteins. Furthermore, GPI-anchored surface proteins act as a protective shield that masks inner cell wall components, such as chitin and β-1,3-glucans, from recognition by plant pattern recognition receptors (PRR), thereby facilitating evasion of host innate immunity. Recently, the GPI-anchoring enzyme ChGPI7 was identified in *C. heterostrophus* as essential for virulence, regulating the localization and stability of cell wall proteins like the putative glycoprotein ChFEM1 (Hu et al., 2025). Deletion of *ChFEM1* similarly impairs infection structure formation, cell wall integrity, and pathogenicity. The study further identified 124 predicted GPI-anchored proteins, offering a rich resource of potential virulence effectors (Hu et al., 2025). Given its conserved role across fungal pathogens, the GPI anchor pathway represents a promising broad-spectrum target for controlling fungal diseases.

## 5 PTM crosstalk

PTM crosstalk represents a fundamental regulatory layer that coordinates multiple modifications on the same or interacting proteins, thereby expanding proreform diversity and enabling higher-order functional complexity.

**Figure 5:**
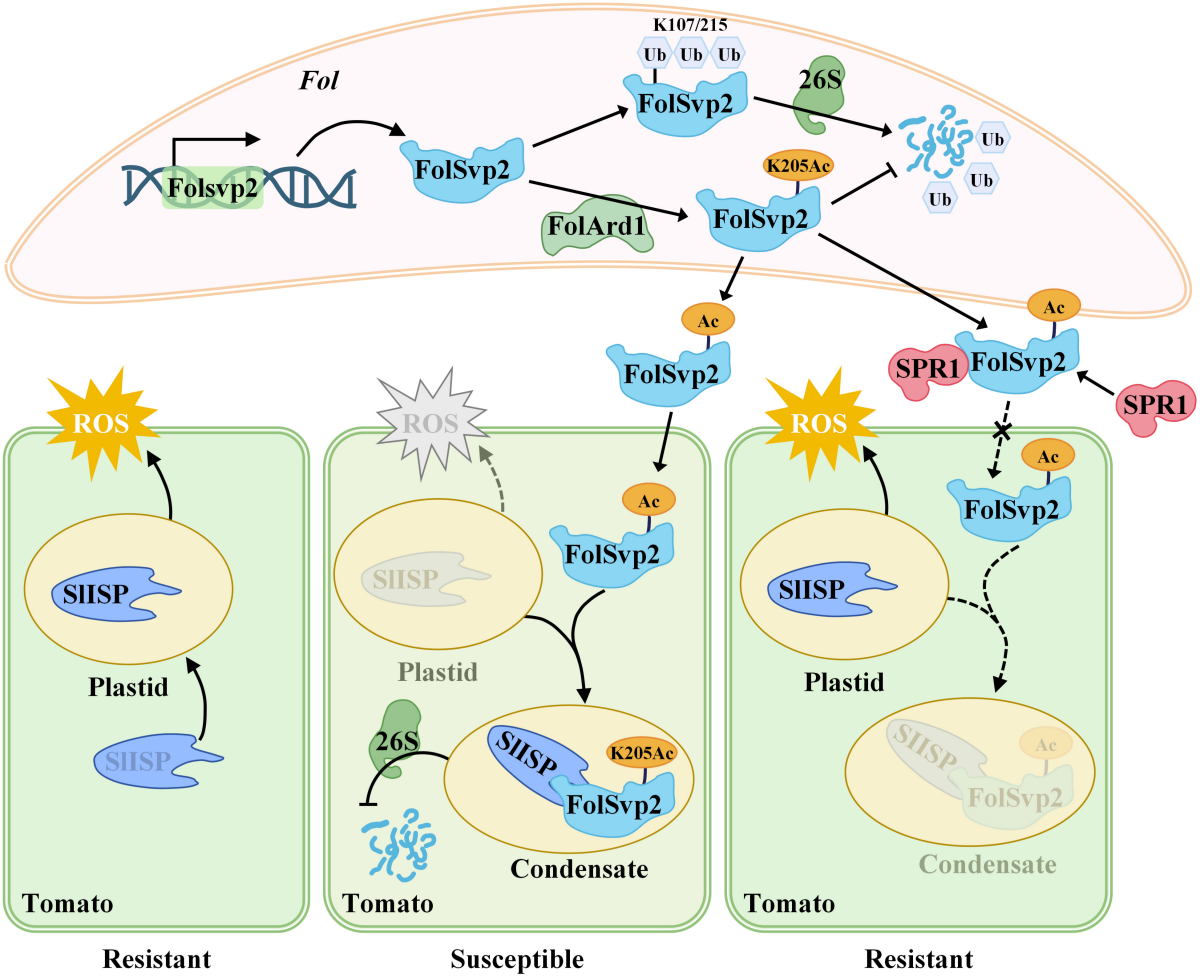
The pathogenesis-associated condensates dynamically assemble through PTM talks in *F. oxysporum*. The fungal effector FolSvp2, secreted by *F. oxysporum*, undergoes PS to sequester the tomato plastid protein SlISP into intracellular condensates. This relocalization suppresses ROS generation and promotes fungal colonization. FolSvp2 function requires acetylation at K205, which protects the effector from ubiquitin-mediated degradation. In defense, tomato secretes the apoplastic protein SlPR1 that directly binds FolSvp2, which prevents its cellular uptake and thereby neutralizes its virulence.

UM often targets proteins for degradation, while AM can stabilize them by blocking UM sites. This antagonistic relationship is exemplified in *F. oxysporum* (Li J. et al., 2022). The effector protein FolSVP1, targets the pathogenesis-related protein PR1 in the host apoplast and hijacks it to enter the host cell nucleus (Li J. et al., 2022). In the host, FolSVP1 undergoes UM at lysine (K167), leading to its degradation and reduced virulence (Li J. et al., 2022). However, the fungal acetyltransferase FolARD1 acetylates the same residue, inhibiting UM and preserving effector toxicity (Li J. et al., 2022). Furthermore, FolSvp2 from *F. oxysporum* forms biomolecular condensates that hijack the host plastidial iron-sulfur protein (SlISP) to suppress ROS and promote infection (Figure 5) (Li et al., 2024). Biomolecular condensate is membraneless organelles and forms through weak multivalent intermolecular interactions of proteins and nucleic acids via phase separation (PS) (Li et al., 2024). FolSvp2 virulence depends on K205 acetylation, which prevents UM-mediated degradation through K107/215, meanwhile the tomato host counteracts this via apoplastic SlPR1, which blocks FolSvp2 cellular entry and neutralize its pathogenicity (Figure 5) (Li et al., 2024). The pathogenesis-associated PS-regulated by PTM, enabling rapidly responses during infection. In plants, the immune regulator NPR1 undergoes redox-sensitive cysteine modifications and nuclear SUMOylation to regulate its condensation, illustrating how redox-SUMO crosstalk modulates immune signaling. The two examples showed that AM and UM compete the same or different residues of the same substrate protein, resulting in antagonistic outcome. Similarly, in plants, the immune regulator NPR1 transitions into cytosolic condensates upon salicylic acid induction, which is mediated by redox-sensitive cysteines and is further modulated by nuclear SUMOylation, illustrating how redox-SUMO crosstalk regulates condensate dynamics and immune signaling (Zavaliev et al., 2020). Above examples indicate that PTMs crosstalk controls effectors virulence, host immune signaling, and stress adaptation during pathogen-host interaction.

Histone modification, particular AM, engage in extensive crosstalk with epigenetic mechanisms. In *N. crassa*, deletion of the histone deacetylase (Hda1) increases histone H3 AM levels while reducing genomic DNA methylation and H3K9me3, suggesting that Hda1 is a critical regulator of epigenome stability (Ebot-Ojong et al., 2025). Besides, in *A. nidulans*, the (complex of proteins associated with Set1) COMPASS subunit CclA and methyltransferase SetA link SUMOylation to histone modifications, highlighting how SUMOylation-deSUMOylation balance, regulated by enzymes like UlpB and UbcN, is crucial for fungal multicellular development (Harting et al., 2013). Here, the intricate crosstalk, where one protein's PTM influence another's, coordinates fungal development and virulence.

A-to-I RNA editing represents a widespread RNA modification that modifies numerous mRNAs in eukaryotes (Xin et al., 2023). Over 40,000 A-to-I editing sites have been detected in perithecia of *N. crassa* and *F. graminearum*. In *F. graminearum*, the deaminase FgTad2-FgTad3-Ame1 complex edit mRNA (Figure 3C) (Feng et al., 2024). Moreover, FgTad3 is regulated by PTMs, including acetylated site (K198) and non-canonical phosphorylated sites (E241), which are specific to sexual stages and essential for ascospore formation (Figure 3C) (Feng et al., 2024). Furthermore, RNA editing technologies like SNAP-ADAR allow precise, reversible modulation of PTM sites (e.g., PM and AM) across signaling proteins, enabling bidirectional control of pathways such as JAK/STAT (Kiran Kumar et al., 2024). RNA editing as a promising tool for PTM interference, with potential therapeutic applications inspired by fungal systems.

Collectively, these examples underscore that the deeper investigation into the dynamic networks of PTM crosstalk provides a comprehensive understanding of fungal pathogenesis. Today, large-scale experimental mapping of PTM crosstalk remains challenging. The integration of multi-omics approaches [e.g., chemical proteomics approach (Zhang N. et al., 2024)] with artificial intelligence and machine learning is poised to accelerate the discovery of PTM networks.

## 6 Conclusion and future perspective

**Figure 6:**
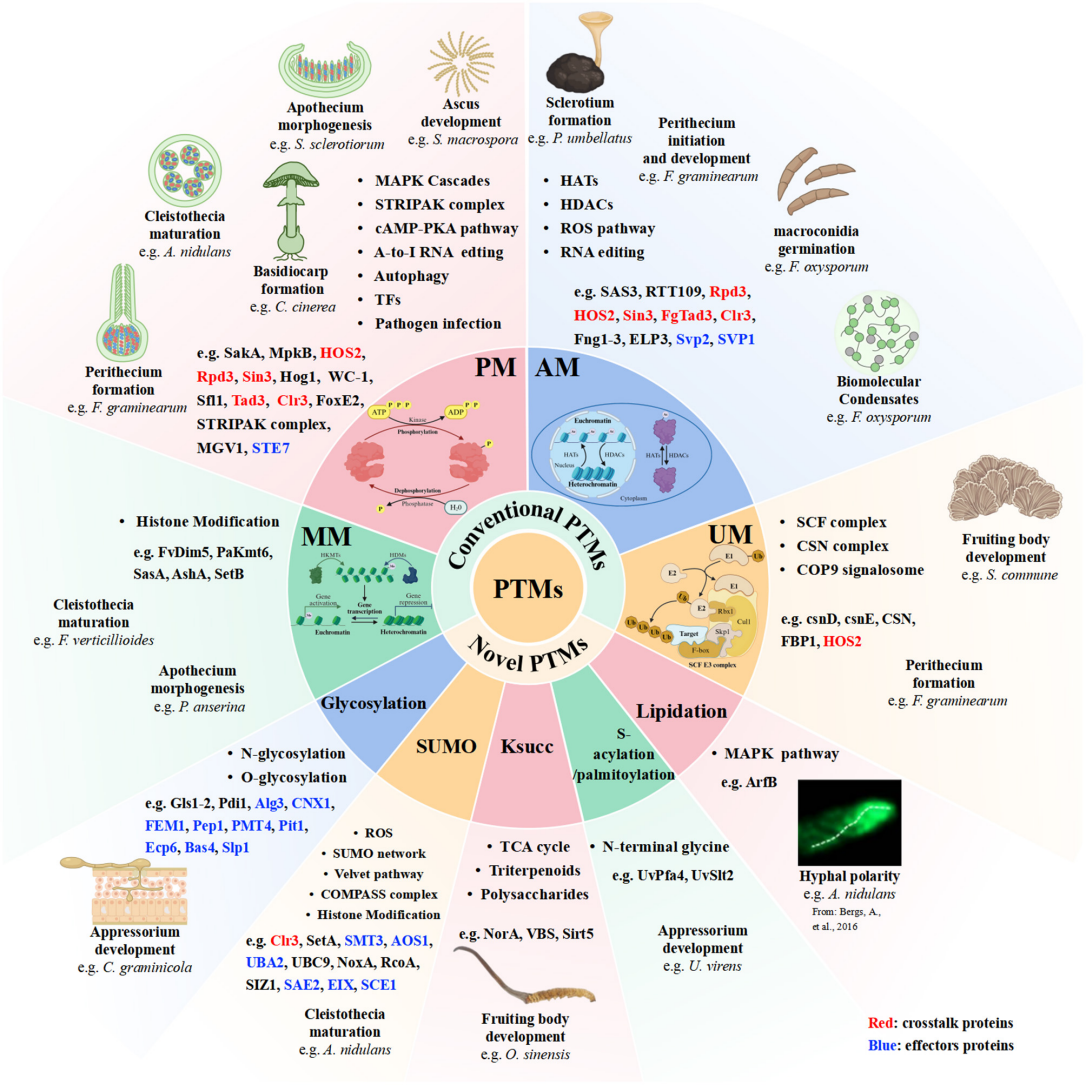
Protein PTMs in sexual reproduction and pathogenesis of filamentous fungi. Proteins involved in PTM crosstalk are highlighted in red, proteins involved in effectors are highlighted in Blue.

Filamentous fungi represent a biologically diverse and metabolically versatile group. Sexual reproduction represents a pivotal phase of the life cycle that is fundamentally linked to bioactive compound biosynthesis. This process is regulated by an intricate regulatory network. PTMs have emerged as a critical mechanism governing the entire sexual development and pathogenesis in filamentous fungi. PTMs expand the functional diversity of the proteome and enable rapid changes at precise time, thereby playing a crucial regulatory role in sexual development, plant–pathogen interactions and environmental adaptation. Studies in model and prevalent phytopathogenic species, such as *N. crassa, Fusarium* spp., and *A. nidulans*, demonstrated that the four most common and novel PTMs, including PM, UM, AM, MM, GlcNAc, SUMOylation, Ksucc, lipidation, S-acylation and GPI, precisely orchestrating fungal sexual development, and pathogenicity. PM is the most extensive PTM in eukaryotic organism and drives signal transduction pathways. UM has emerged as a key regulator of fungi-plant interaction, and dynamically crosstalks with other PTMs to orchestrate immune signaling. Its structural complexity arising from diverse ubiquitin chain architectures further adds to the intricacy of UM. AM also plays significant regulatory role in fungal defense mechanisms. The crosstalk among different PTMs requires further investigation Future research will focus on (1) the dynamics of PTMs levels and response mechanisms under diverse stress conditions; (2) advancing technologies for large-scale identification of PTM targets, either through integrated proteomics or bioinformatics approaches, thereby revealing new targets for fungicide; (3) uncovering key enzymes regulating PTMs as potential plant diseases intervention; (4) developing innovative technologies for discovering novel PTMs, such as MS-based proteomics analysis, bioinformatic approaches and chemical proteomics strategies that rely on the design of specific chemical probes to functionally characterize PTM-modified proteins; (5) knowledge of PTM crosstalk at high spatiotemporal resolution is essential for fully understanding how the interplay among PTMs, advancing the large-scale identification of PTM crosstalk through the integration of multiomics approach with machine learning methods and artificial intelligence approach; (6) exploiting genome editing tools to precisely manipulate specific PTM sites for phytopathogenic fungi control and strain breeding. E.g., CRISPR/Cas-derived base editors and prime editors offer a powerful means to generate precise point mutations at targeted genomic sites that enables modulation of multifunctional protein interactions for pathogen control and strain breeding. RNA editing further provides an effective tool for modulating protein function by specifically targeting regulatory PTM sites. Protein PTMs in sexual reproduction and pathogenesis of filamentous fungi were shown in Figure 6. Summary of roles of PTM in regulating sexual development and plant fungal pathogenicity is shown in Table 1. The roles of PTM in modulating effectors and infection-associated proteins in regulating plant fungal pathogenicity is shown in Table 2.

**Table 1:** Summary of the roles of different PTMs in regulating sexual development and plant fungal pathogenicity.

**PTMs type**	**Species**	**Protein name**	**Modification Sites**	**Enzyme(s) involved**	**Function**	**Ref(s)**
PM	*Aspergillus fumigatus*	CpcB	-	MAPK	Regulates sporulation, conidial germination, cell wall integrity, drug sensitivity, and virulence	Zhang et al., 2016
	*Aspergillus nidulans*	CpcB	-		Regulates sexual development, sporulation, and toxin synthesis	Zhang et al., 2016
		MkkB	-	MAPKKK	Regulates hyphal fusion, conidiation, and cleistothecium formation	Frawley et al., 2018
		MpkB	-	MAPK	Regulates cleistothecium formation	
	*Botrytis cinerea*	BcSAK1	The TDY motif		Regulates the responses to osmotic, oxidative, and fungicide stress, sporulation, sclerotial formation, and penetration infection	Segmüller et al., 2007
	*Bipolaris oryzae*	srm1	The TGY motif		Affects sensitivity to osmotic and UV stresses	Moriwaki et al., 2006
	*Candida albicans*	Asc1	-	MAPK	Regulates adhesion, invasive growth, and virulence	Zhang et al., 2016
	*Cochliobolus carbonum*	HOS2^*^	-	SNF1	Activates its deacetylase activity, regulates pathogenicity and tolerance to HC-toxin	Zhang X. et al., 2024
	*Cochliobolus heterostrophus*	Rpd3^*^	S421	-	Regulates the infection ability and pathogenicity of the strain, stabilizes the structure of the Rpd3-Sin3 complex, and activates HDAC enzyme activity	Fan et al., 2025
		ChPho23	T227, S314	CaMK	Stabilizes the ternary structure of the Rpd3-Sin3 complex and mediates nuclear localization of the complex, regulating conidial germination and infectious hyphal extension in the strain	
		Sin3^*^	T358	CaMK	Mediates interaction with ChCrz1, integrates calcium signaling, and regulates infection peg formation	
		ChSds3	S189	CDK	Mediates binding to Sin3, enhances the HDAC enzyme activity of Rpd3, regulates the specificity of the nitrosative stress response, and indirectly affects the formation of infection pegs	
		ChHog1	T171/Y173	MAPKK	Mediates the nuclear import of ChHog1 and regulates the transcription of osmotic and nitrosative stress-related genes to counteract host reactive nitrogen stress, while also determining the interaction capability with the Rpd3-Sin3 complex. Additionally, it regulates appressorium formation and the penetration ability of infection pegs into the host	
		ChCrz1	S256/S260	Calcineurin	Regulates the nucleocytoplasmic shuttling of ChCrz1, determines the interaction specificity with the Rpd3-Sin3 complex, mediates the cross-regulation between calcium signaling and nitrosative stress, and additionally, maintains the polarized growth of the strain's infectious hyphae	
	*Coprinopsis cinerea*	CcSakA	T170, Y172	MAPKK (Pbs2)	Regulation of stipe elongation through the HOG pathway	Zhao et al., 2022
	*Cryptococcus neoformans*	Gib2	-	PKA	Regulates virulence factors such as capsule formation and melanin synthesis	Zhang et al., 2016
	*Fusarium graminearum*	cpk1/2	-		Regulates perithecia formation	Hu et al., 2014
		FAC1	-	-	Regulates pathogenicity by affecting infection structure formation and DON synthesis	
		MGV1	T174, G175, T176	MAPKK	Regulates female fertility; the mutant strain fails to form mature perithecia and ascospores	Hou et al., 2002
		Gip1	-	-	Regulates perithecium formation	Ding et al., 2025
		FgSfl1	S223, T452, S559	PKA	Regulates ascocarp morphogenesis and ascospore release via the cAMP/PKA pathway	Gong et al., 2021
		FgTad3^*^	E8, E241	tRNA modification enzyme	Regulates ascospore formation via A-to-I RNA editing	Feng et al., 2024
	*Mycosphaerella graminicola*	MgSlt2	T190, Y192, T195	MAPKK	Regulates colonization and invasive growth	Mehrabi et al., 2006a
		MgHog1	-		Regulates osmotic stress response, melanin synthesis, and sensitivity to phenylpyrrole and dicarboxylic acid fungicides	Mehrabi et al., 2006b
	*Magnaporthe grisea*	MPS1	T190, Y192	MAPKK (Mkk2)	Essential for conidiation, appressorial penetration, and infection	Xu et al., 1998
		PMK1	-	MAPKK (Ste7)	Regulates appressorium formation, infectious growth in plant cells, and the expression of immune-suppressing effector genes	Liu W. et al., 2021
	*Neurospora crassa*	MOB3	N-terminal domain	STRIPAK	Mediates karyogamy via the STRIPAK complex	Gordon et al., 2011
	*Neurospora circadian*	WC-1	zinc finger region	PKC, GSK-3	Probably regulates the nuclear location of WC-1 for controlling fungal sexual development	He et al., 2005
	*Podospora anserina*	PaKmt6	H3K27me3	PRC2	Regulates transcriptional, fruiting body morphogenesis, spore germination, and production of male gametes	Carlier et al., 2021.
	*Schizophyllum commune*	Hom2	Four RRXS motifs	PKA	Initiates early primordium formation	Pelkmans et al., 2017
	*Sordaria macrospora*	CLA4	S685	STE20	Regulates the formation of ascus and ascospore formation via he STRIPAK complex	Märker et al., 2020
		DBF2	S106	Dbf2	Regulates cell division during sexual spore formation via the STRIPAK complex	Shariatnasery et al., 2024
		Gul 1	S216	PP2A	Regulates the fruiting body maturation via the STRIPAK complex	Stein et al., 2020
	*Sclerotinia sclerotiorum*	SsFoxE3	-	-	Activate SsAtg8transcription, regulates appressorium formation and fugal pathogenicity, and regulates autophagy and ROS homeostasis	Jiao et al., 2022
		SsAtg8	-	-	Mediates autophagosome assembly and pathogenicity	
		SsFoxE2	S182, S223, S261, S329	Fus3	Affects ubiquitination balance and ascocarp formation via autophagy	Zhu et al., 2025
	*Ustilago maydis*	Rak1	-	STE20	Regulates the sensitivity to cell wall stress, cell fusion, mating tube formation, and virulence	Zhang et al., 2016
		Crk1	-	CDK	Regulates mating, hyperpolarized growth and infection virulence	Garrido et al., 2004
		Clr3^*^	S114, T289	Kpp6	Relieves its autoinhibitory N-terminus, enhances deacetylation efficiency, and strengthens binding to Swi6 for improved targeting of telomeric and mating-type chromatin	Zhang X. et al., 2024
		Hos2^*^	S207, T312	Kpp2	Relieves the autoinhibitory conformation to activate its deacetylase activity	
	*Verticillium dahliae*	VdRACK1	-	-	Regulates the root penetration and colonization capabilities	Zhang et al., 2016
AM	*Aspergillus fumigatus*	Sas3	H3K9, H3K14	HATs and HDACs	Regulates strain growth, conidia formation, cell wall integrity, and virulence	Wang et al., 2024
		RTT109	H3	HATs and HDACs	Regulates conidiation, cell wall integrity, and drug resistance	Zhang X. et al., 2024
	*Aspergillus flavus*		H3K9, H3K14	HATs and HDACs	Regulates spore germination, hyphal penetration ability, aflatoxin synthesis, and oxidative stress response	
	*Beauveria bassiana*	Rpd3	H3K9, H3K14, H4K16	HDACs	Regulates hyphal growth, spore formation, environmental tolerance, appressorium formation, and evasion of host immunity to enhance colonization efficiency	Zhang Y. et al., 2024
	*Botrytis cinerea*	Rpd3	H3K9, H3K14, H4K5, H4K8, H4K12, H4K16	HDACs	Regulates strain growth, conidiation, appressorium formation, hyphal infection expansion capacity, stress adaptation, and suppression of host defense responses	Zhang X. et al., 2024
	*Cochliobolus carbonum*	HOS2^*^	H3K9, H3K14, H4K16	HDACs	Regulates carbon metabolic switching, extracellular depolymerase expression, pathogenicity, conidial development, and dimorphic transition	Zhang X. et al., 2024
	*Cochliobolus heterostrophus*	Rpd3^*^	H3K9, H3K14, H4K8, H4K16	HDACs	Regulates histone H3/H4 deacetylation, affecting conidial germination, nitrosative stress response, infection penetration ability, appressorium formation, and toxin synthesis	Fan et al., 2025
		Sin3^*^	-	Rpd3	Regulates nucleocytoplasmic shuttling ability, impacting Rpd3-Sin3 complex formation, nitrosative stress response, and pathogenicity	
	*Fusarium graminearum*	FgTad3^*^	K198	tRNA modification enzyme	Regulates ascospore formation via A-to-I mRNA editing	Feng et al., 2024
		FgGCN5	H3K9, H3K14, H3K18, H3K27	HATs and HDACs	Regulates perithecium formation and ascospore production	Kong et al., 2018
		FgSAS3	H3K4, H3K14			
		RTT109	H3K56			Schneider et al., 2006
		Fng1	H4		Regulates H4 acetylation and perithecium initiation	Jiang et al., 2020
		FgEsa1				
		Fng2	H4		Interacts with the Rpd3 HDAC complex to modulate H3/H4 deacetylation, regulating virulence and perithecium development	Xia et al., 2024
		Fng3	H4		Promotes H3 acetylation and interacts with the Rpd3 HDAC complex to deacetylate H4, thereby maintaining an acetylation-deacetylation equilibrium essential for asci and ascospore maturation	Xu et al., 2023
		ELP3	H3K14		Promotes H3 acetylation, regulating perithecium and ascospore development	Lee et al., 2014
	*Fusarium oxysporum*	FolSvp2	K205	AT and HDAC	Prevents ubiquitin-mediated degradation, hijacking SlISP to suppress host ROS production and promote infection	Li et al., 2024
	*Monascus* spp.	RTT109	H3	HATs and HDACs	Regulates hyphal morphogenesis, environmental adaptation, and pathogenicity to the host	Zhang X. et al., 2024
	*Magnaporthe oryzae*	MoSAS3	H3K14	HATs and HDACs	Regulates strain growth, spore germination, appressorium formation, and pathogenicity	Dubey et al., 2019
		Rpd3	H3K9, H3K14, H4K5, H4K8, H4K12, H4K16	HDACs	Regulates strain growth, conidiation, appressorium formation, hyphal infection expansion capacity, stress adaptation, and invasive pathogenicity	Zhang X. et al., 2024
		SIR2	H3K9, H4K16	HDACs	Regulates conidial morphogenesis and germination, appressorium formation, evasion of host immune recognition, and enhances stress tolerance	
		MoJMJC	L287	SIR2	Regulates the transition of host conidia to appressoria, host immune defenses, the adaption to host ROS defense system, and histone H3K4 demethylase activity	
	*Ustilago maydis*	Clr3^*^	L417	Esa1	Suppresses target enzyme activity and deacetylation efficiency, sustains intracellular deacetylase homeostasis	
			H3K9	HDACs	Regulates mating-type gene silencing and infection morphological switching	
			H4K20	HDACs	Interferes with host immune defenses	
		Hos2^*^	L158	Gcn5	Autonomous acetylation modification	
			H3K9	HDACs	Regulates the transition from the yeast-like budding to the invasive hyphae, mediates the proliferation of infectious hyphae, and enables evasion of host immune recognition	
MM	*Aspergillus flavus*	AshA, SetB	H3K36me1	HMTase	Regulates sexual development-related genes, such as steA	Zhuang et al., 2022
	*Aspergillus nidulans*	SasA	-	SAM	Regulates perithecium development	Gerke et al., 2012
	*Fusarium verticillioides*	FvDim5	H3K9me3	KHMTase	Regulates perithecium formation and sexual development	Gu et al., 2017
	*Podospora anserina*	PaKmt6	H3K27me3	PRC2	Regulates fruiting body morphogenesis and spermatia production	Carlier et al., 2021.
UM	*Aspergillus nidulans*	csnD, csnE	-	CSN	Regulates cleistothecia or fruiting bodies maturation	Busch et al., 2003
	*Gibberella zeae*	FBP1	-	E3	Regulates perithecium and ascospore formation	Han et al., 2007
	*Ustilago maydis*	Hos2^*^	L89	Siz1	Mediates nuclear localization, protein turnover regulation and adaption during infection	Zhang X. et al., 2024
Ksucc	*Aspergillus flavus*	NorA	K370	Succinyltransferase	Regulates sclerotial yield, colonization capacity, sporulation, and AFB1 biosynthesis	Ren et al., 2018
		VBS	K135	STs and DSs	Catalyzes the key oxidation reactions in aflatoxin synthesis, regulates sclerotial development, environmental adaptation, the survival and virulence	Wang et al., 2023
	*Magnaporthe oryzae*	Sirt5	-	-	Regulates appressorium formation, invasive hyphal expansion, and antioxidant response	Ren et al., 2024
SUMOylation	*Aspergillus nidulans*	AosA/UbaB	-	E1	Regulates cleistothecium formation	Harting et al., 2013
		UbcN	-	E2		
		UlpA	-	DeSUMOylase		
		SetA	-	AosA/UbaB, UbcN		
		NoxA	-	NoxR and RacA	Regulates sexual development at microcleistothecia stage and ascospore formation	Lara-Ortíz et al., 2003
		RcoA	-	COMPASS	Regulates cleistothecium formation	Harting et al., 2013
		SumO	-	SUMOylase	Regulates cleistothecium formation	Harting et al., 2013
	*Fusarium oxysporum f*. sp. *niveum*, Fon	SIZ1	-	-	Recognizes and binds to target proteins, mediates the covalent attachment of SMT3, and essential for the survival	Azizullah et al., 2023
	*Ustilago maydis*	Clr3^*^	L352	Siz1	Mediates nuclear localization and enhances its binding affinity to histone H3	Zhang X. et al., 2024
GlcNAc	*Fusarium oxysporum*	Gnt2	-	Glycosyltransferases	Regulates colonization, expansion, and immune evasion	López-Fernández et al., 2013
	*Mycosphaerella graminicola*	GPI-APs	-		Regulates chitin-binding affinity and morphological integrity during spore germination	Liu W. et al., 2021
		Alg3	-		Regulates immune evasion	Liu W. et al., 2021
		MgAlg2	-		Regulates hyphal invasive growth and cell wall integrity	Motteram et al., 2011
	*Magnaporthe oryzae*	Gls1	N497		Regulates invasive hyphal growth and conidiation	Goto, 2007
		Gls2	-		Regulates conidial development and virulence	Goto, 2007
		GTB1	-		Regulates sporulation, pathogenicity, and cell wall integrity	
		Cnx1	-		Regulates sporulation, appressorium colonization, and pathogenicity	
		Slp1	N48, N104, N131		Regulates the expansion of infectious hyphae through competitively binding to host chitin, attenuating the host's innate immunity	Chen X. L. et al., 2014
		Bas4	N36	N-glycosyltransferases	Regulates extracellular stability, and immunomodulatory activity	Chen X. L. et al., 2014
	*Ustilago maydis*	Pdi1	N89, N307	Glycosyltransferases	Alleviates endoplasmic reticulum stress, and promotes effectors secretion and infectivity	Marín-Menguiano et al., 2019
S-acylation	*Ustilaginoidea virens*	UvSlt2	-	Acyltransferases	Regulates spore formation and infectivity	Duan et al., 2024

^*^Presents proteins involved in PTMs crosstalk.

CpcB, Phycocyanin beta subunit; MkkB, Mitogen-activated protein kinase kinase B; MpkB, Mitogen-activated protein kinase B; SAK1, Stress-Activated Protein Kinase 1; srm1, Suppressor of ras mutation 1; Asc1, Activator of stress response 1; Gib2, G protein beta-like subunit 2; cpk1/2, Calcium-dependent protein kinase 1/2; FAC1, Forkhead-associated domain-containing protein 1; MGV1, Magnaporthe grisea Virulence homolog 1; Gip1, G protein-interacting protein 1; Sfl1, Suppressor of flocculation 1; Tad3, Telomere-associated protein 3; Slt2, Stress-activated protein kinase Slt2; Hog1, High-osmolarity glycerol response kinase 1; MPS1, Monopolar spindle 1 kinase; PMK1, Pathogenicity mitogen-activated protein kinase 1; MOB3, Mps one binder kinase activator-like 3; WC-1, White Collar-1; Kmt6, Lysine methyltransferase 6; Hom2, Homoserine dehydrogenase 2; CLA4, Cla4 serine/threonine kinase; DBF2, Dbf2 serine/threonine kinase; Gul1, Galactose utilization-like protein 1; FoxE3, Forkhead box transcription factor E3; Atg8, Autophagy-related protein 8; FoxE2, Forkhead box transcription factor E2; Rak1, Receptor-associated kinase 1; Crk1,Cyclin-dependent kinase-like 1; RACK1, Receptor for Activated C Kinase 1; GCN5, General control non-repressible 5; SAS3, Something About Silencing 3; RTT109, Regulator of Ty1 transposition 109; Fng1-3, Fucosyltransferase-like protein 1/2/3; ELP3, Elongator complex protein 3; Svp2, Stolonization-related protein 2; AshA, Histone lysine methyltransferase AshA; SetB, SET domain-containing protein B; SasA, Sensor histidine kinase SasA; Dim5, Dim5-like histone methyltransferase; csnD/E, COP9 signalosome subunit D/E; FBP1, Fructose-1,6-bisphosphatase 1; NorA, Norfloxacin resistance protein A; VBS, Virulence-associated binding protein S; Sirt5, Sirtuin 5; AosA/, Activator of stress response A; UbaB, Ubiquitin-activating enzyme B; UbcN, Ubiquitin-conjugating enzyme N; UlpA, Ubiquitin-like-specific protease A; SetA, SET domain-containing protein A; NoxA, NADPH oxidase A; RcoA, Repressor of carbon metabolism A; SumO, Small ubiquitin-related modifier; SIZ1, SAP and Miz domain-containing protein 1; Gnt2, Gluconate transporter 2; GPI-APs, Glycosylphosphatidylinositol-anchored proteins; Alg2/3, Asparagine-linked glycosylation 2/3; Gls1/2, Glutaminase 1/2; GTB1, Glycosyltransferase B1; Cnx1, Calnexin 1; Slp1, Secreted lysophospholipase 1; Bas4, Blastospore-associated protein 4; Slt2, Stress-activated protein kinase Slt2; HC-tox, Helminthosporium carbonum toxin.

**Table 2:** Roles of different PTMs in modulating effectors and infection-associated proteins for facilitating entry and infection processes of plant pathogens fungi.

**PTMs type**	**Pathogen**	**Host**	**Effectors and infection-associated proteins**	**Modification sites**	**Enzymes involved**	**Response**	**References**
PM	*Colletotrichum higginsianum*	*Arabidopsis thaliana*	ChSTE7	-	MAPKK	Regulates appressorium development, and invasive hyphal growth	Yuan et al., 2016
AM	*Fusarium oxysporum*	*Solanum lycopersicum*	FolSvp1	K167	AT and HDAC	Suppresses plant immunity and promotes infection	Li J. et al., 2022
			FolSvp2	K205	AT and HDAC	Hijacking the host plastid iron-sulfur protein SlISP to inhibit host ROS production, thereby inhibiting plant immunity and promoting infection	Li et al., 2024
GlcNAc	*Colletotrichum graminicola*	*O. sativa*	ALG3	-	Glycosyltransferases	Encodes an α-1,3-mannosyltransferase responsible for the biosynthesis of N-glycans, which is required for glycosylation of effector proteins, influencing appressorium activity and effector secretion	Mei et al., 2023
		*Zea mays*	CNX1	-	Glycosyltransferases	Regulates colonization, affecting effectors secretion	
	*Cochliobolus heterostrophus*	*Zea mays*	ChFEM1	-	Gnt2	Required for appressoria development for penetrating the host cuticle	Hu et al., 2025
	*Ustilago maydis*	*Zea mays*	Pep1	-	Glycosyltransferases	Suppresses the host's peroxidase activity, thereby blunting the ROS burst and ultimately hinders the plant's defense response	Doehlemann et al., 2009
			PMT4	-	Glycosyltransferases	O-glycosylation pathway gene PMT4 required for appressorium appressorium differentiation and penetration	Fernández-Álvarez et al., 2012
			Pit1	-	Glycosyltransferases	Maintains cellular integrity and regulates nutrient acquisition from the host, suppresses the host's basal defense response, thereby enabling its own proliferation and spread in host	
	*Cochlibolus heterostrophus*	*Oryza sativa, Hordeum vulgare*	GPI7	-	Glycosyltransferases	Regulates appressorial cell wall integrity, maintains turgidity and penetration	Hu et al., 2025
	*Fusarium oxysporum*	*Solanum lycopersicum*	GNT2	-	Glycosyltransferases	N-glycosylation pathway proteins that required for appressoria development to penetrate the host cuticle	López-Fernández et al., 2013
	*Cladosporium fulvum*	*Solanum lycopersicum*	Ecp6	-	Glycosyltransferases	Competitively inhibits the host immune receptor's ability to bind chitin	Sánchez-Vallet et al., 2013
	*Magnaporthe oryzae*	*Oryza sativa*	Bas4	N36	N-glycosyltransferases	Maintains the integrity of invasive hyphae and enhances the pathogenicity	Mosquera et al., 2009
			Slp1	N48, N104, N131	Glycosyltransferases	Regulates the expansion of invasive hyphae, competitively binding to host chitin to weaken its innate immunity	Chen X. L. et al., 2014
SUMOylation	*Fusarium oxysporum*	*Solanum lycopersicum*	SMT3	-	SUMOylation pathway enzymes	Required of hyphal differentiation and growth, cell wall integrity, spore formation and germination, colonization ability, and pathogenicity	Azizullah et al., 2023
			AOS1, UBA2	-	SUMOylation pathway enzymes	Activates SMT3, enhances stress tolerance, inhibits abnormal apoptosis, and governs the strain's infective	
			UBC9	-	SUMOylation pathway enzymes	Mediates SMT3 transfer from E1 to target proteins, modulates hyphal growth and spore morphology, ensuring cell wall homeostasis, enhancing stress tolerance, balancing apoptosis and autophagy, and facilitating late-stage host adaptation	
	*Trichoderma viride*	*Solanum lycopersicum*	EIX	-	SUMOylation pathway enzymes	Reduces tomato SUMO transcript levels, leading to the activation of defense response	Sharma et al., 2021
	*Plectosphaerella cucumerina*	*Arabidopsis thaliana*	SAE2, SCE1	-	SUMOylation pathway enzymes	Affects the turnover rates of SAE2 and SCE1, resulting in a reduced levels of both free SUMO and SUMO conjugates	Castaño-Miquel et al., 2017

STE7, MAPKK STE7-like protein; SVP1, Short Vegetative Phase 1; Svp2, Short Vegetative Phase 2; ALG3, Asparagine-Linked Glycosylation 3; CNX1, Calnexin 1; Pep1, Pathogenicity Effector Protein 1; PMT4, Protein O-Mannosyltransferase 4; Pit1, Pathogen-Induced Transmembrane Protein 1; Ecp6, Extracellular Protein 6; Bas4, Basidiospore Formation 4; Slp1, Secreted Lysin Motif Protein 1; SMT3, Small Ubiquitin-like Modifier 3; AOS1, Activating Enzyme Subunit 1; UBA2, Ubiquitin-like Modifier Activating Enzyme 2; UBC9, Ubiquitin-Conjugating Enzyme 9; EIX, Ethylene-Inducing Xylanase; SAE2, SUMO-Activating Enzyme Subunit 2; SCE1, SUMO-Conjugating Enzyme 1; GnT, GlycanN-acetylglucosaminylTransferase; GPI, Glycosylphosphatidylinositol.
